# A Correlation-Decoupled Interval Belief Rule Base for Interpretable Cross-Condition Bearing Fault Diagnosis

**DOI:** 10.3390/e28080939

**Published:** 2026-08-21

**Authors:** Xingchi Yan, Yan Yu, Ning Li

**Affiliations:** School of Computer Science and Information Engineering, Harbin Normal University, Harbin 150025, China; 2024300741@stu.hrbnu.edu.cn (X.Y.);

**Keywords:** cross-condition bearing fault diagnosis, correlation decoupling, interval belief rule base, evidential reasoning, complex systems

## Abstract

Cross-condition bearing fault diagnosis requires models that remain reliable under load-induced distribution shifts while providing transparent and traceable reasoning. Conventional belief rule bases (BRBs) may repeatedly use correlated vibration evidence during inference, and their Cartesian-product rule construction can rapidly increase rule-base complexity. This study proposes a correlation-decoupled interval belief rule base (CD-IBRB) for cross-condition bearing fault diagnosis. Seven diagnostically relevant time-domain features are selected using XGBoost and transformed into a less-correlated feature space through a Kendall-rank-correlation-guided matrix estimated exclusively from the source training data. Attribute-wise referential points and intervals are then constructed from the transformed training attributes, allowing the rule base to grow additively rather than combinatorially. Initial belief distributions are obtained from interval-level class distributions. The projection covariance matrix adaptation evolution strategy (P-CMA-ES) jointly optimizes the belief degrees, rule reliabilities, and rule weights, while evidential reasoning aggregates the activated interval rules to produce the final diagnostic result. In the primary cross-load bearing experiment, CD-IBRB achieved an accuracy of 0.9702 and a macro-averaged F1 score of 0.9703. It outperformed the strongest BRB variant and data-driven baseline by 7.70 and 6.10 percentage points in accuracy, respectively. Ablation experiments confirmed that removing parameter optimization or attribute decoupling reduced accuracy to 0.9053 and 0.9303, respectively. Additional cross-load and noise-injection experiments further demonstrated the stability of CD-IBRB under load shifts and input disturbances. Across five public multiclass datasets, CD-IBRB achieved a mean accuracy of 0.9004 and consistently outperformed the compared BRB variants. These results demonstrate that CD-IBRB provides a compact, uncertainty-aware, and traceable framework for cross-condition bearing fault diagnosis.

## 1. Introduction

With the continued development of modern industrial equipment toward higher speeds, greater complexity, and increased intelligence, the operating condition of critical rotating components, particularly bearings, has become essential to the safety, reliability, and service life of mechanical systems. During long-term operation, bearings are susceptible to load fluctuations, speed variations, lubrication degradation, and external disturbances. Localized damage or performance deterioration may intensify vibration, reduce transmission accuracy, and even cause unexpected system shutdowns. Therefore, developing accurate, robust, and interpretable bearing fault diagnosis models is crucial for ensuring the safe operation of complex engineering systems [1].

Existing fault diagnosis methods can generally be classified into knowledge-driven, data-driven, and hybrid knowledge-and-data-driven approaches [2,3]. Knowledge-driven methods primarily rely on expert experience, fault mechanisms, and rule-based reasoning, thereby providing transparent diagnostic processes and strong interpretability. Li et al. proposed a hierarchical object-oriented Bayesian network-based fault diagnosis method that constructs system-level diagnostic models by reusing and inheriting standardized Bayesian network fragments, thereby addressing repetitive and inconsistent modeling in large-scale building energy systems under incomplete and uncertain information [4]. Dou et al. proposed an intelligent rule-based fault diagnosis method integrating empirical mode decomposition, dimensionless features, the MLEM2 rule-induction algorithm, and an improved rule-matching strategy, which addresses weak fault-feature extraction from nonstationary bearing signals and the conflicting or unmatched rules encountered in rotating machinery diagnosis [5]. Zhou et al. experimentally investigated vibration-based bearing fault diagnosis using kurtosis, root-mean-square vibration velocity, and resonance demodulation spectra, and refined the corresponding diagnostic rules to reduce false diagnoses caused by rigid thresholds and ambiguous engineering criteria [6]. Yu et al. proposed a model-based sensor fault diagnosis and fault-tolerant control scheme that combines recursive least squares and unscented Kalman filtering with open-circuit-voltage and capacity residuals, addressing the detection, isolation, and estimation of voltage- and current-sensor faults in electric-vehicle batteries across different state-of-charge intervals [7]. Li et al. developed a semi-supervised framework for bearing fault diagnosis that combines wavelet packet decomposition and information-entropy features with CNN-based representation learning, enhanced attention, and XGBoost classification. This framework reduces reliance on extensively labeled samples while improving diagnostic generalization under limited-data conditions [8]. Peng et al. developed a knowledge graph completion framework for rolling bearing fault diagnosis that integrates heterogeneous multimodal information with semantic relationships among fault entities. This approach enables more effective use of multisource diagnostic evidence and improves fault identification accuracy under complex operating conditions [9]. Sotnykov et al. conducted a mechanism-based failure analysis of hinge-lever mould oscillator bearings in a continuous casting machine. By combining kinematic and dynamic-load analysis with roundness measurements, optical microscopy, and scanning electron microscopy of an in-service 22316E bearing, they identified false brinelling followed by abrasive wear as the principal damage mechanism. The study further showed that non-uniform dynamic loading and inadequate lubrication conditions contributed to the formation of dents and wear traces on the bearing races [10]. However, their effectiveness under complex operating conditions and uncertain environments is often constrained by incomplete domain knowledge and subjective expert judgment.

In contrast, data-driven methods automatically learn the nonlinear mapping between monitoring signals and health states from large-scale operational data, thereby reducing their dependence on explicit physical models and manually defined diagnostic rules. Fu et al. proposed a motor bearing fault diagnosis framework that combines an auxiliary-classifier generative adversarial network with a Transformer architecture. The method alleviates the shortage of labeled fault samples and improves feature extraction and classification performance for small datasets [11]. Su et al. proposed a bearing fault diagnosis framework that integrates data reconstruction with hierarchical recurrent meta-learning, thereby improving cross-condition generalization when only limited labeled samples are available [12]. Wang et al. developed a bearing fault diagnosis framework that integrates vibration and acoustic measurements through a one-dimensional convolutional neural network, thereby improving diagnostic accuracy and robustness to noise compared with single-modality approaches [13]. Zhang et al. reviewed representative deep learning methods for bearing fault diagnosis and identified limited labels, class imbalance, noisy measurements, cross-condition generalization, and insufficient interpretability as major barriers to their practical application [14]. Li et al. developed a weighted multidimensional feature selection and fusion method that combines single-feature SVM evaluation, correlation analysis, and PCA-based weighted loading evaluation, followed by PSO-SVM classification. The method reduces redundant and irrelevant information in high-dimensional feature sets while preserving sensitive features with clearer physical meanings, thereby improving the identification of bearing fault types and severity levels [15]. Xu et al. proposed a cross-sensor self-contrastive learning method for nondestructive fault detection of heavy locomotive motors. By integrating inter-sensor and intra-sensor contrastive learning with multisource envelope-spectrum feature fusion, the method addresses the limited availability of labeled samples and the insufficient utilization of complementary information across sensors [16]. Jin et al. developed a dual correlation graph knowledge distillation network for bearing remaining useful life prediction. The method combines dynamic graph representation learning, multilevel spatiotemporal knowledge distillation, and uncertainty quantification to improve computational efficiency and prediction reliability while accounting for vibration noise and model variability [17]. However, their performance generally depends on sufficient high-quality labeled data, while their opaque internal decision-making processes make it difficult to satisfy the interpretability requirements of safety-critical engineering applications.

To overcome these limitations, hybrid knowledge-and-data-driven methods integrate expert knowledge with observed samples, enabling them to balance diagnostic accuracy and model interpretability while effectively handling uncertainty in complex systems. Consequently, these methods have been widely applied to fault diagnosis tasks. Li et al. developed an interpretable bearing fault diagnosis framework that combines a nested U-Net waveform segmentation model with a class-weighted loss and physics-informed denoising constraints. The method addresses the reliance of conventional signal processing on expert knowledge, the difficulty of extracting weak fault impulses under strong noise, and the limited interpretability of deep learning models [18]. Liao et al. proposed a classifier-guided neural blind deconvolution framework that integrates time- and frequency-domain neural filters with a physics-informed loss function. This method resolves the incompatibility between conventional blind deconvolution and downstream classifiers while improving fault-sensitive feature extraction under noisy conditions [19]. Chen et al. proposed a bearing fault diagnosis and interpretation method based on frequency-temporal logic, in which Bayesian optimization and Bayesian neural networks are employed to determine the logical structure and parameters. The method addresses the opaque decision-making process of conventional intelligent models and enables interpretable fault diagnosis under noisy and variable-speed conditions [20]. He et al. developed a cross-machine transfer diagnosis network that combines a modulated differentiable short-time Fourier transform with a physics-informed balanced spectrum quality constraint. This approach addresses insufficient time–frequency feature extraction under speed fluctuations and reduces domain discrepancies caused by limited fault samples from freight-train wheelset bearings [21]. Ni et al. proposed a physics-informed residual network that integrates modal-property-based feature generation, domain conversion, and dual-channel residual learning. The method reduces dependence on large-scale labeled data and improves diagnostic robustness against distribution shifts caused by variable operating speeds, loads, and noise [22]. Zhou et al. developed a probabilistic Bayesian deep learning framework that quantifies epistemic and aleatoric uncertainties in diagnostic predictions. The method improves the trustworthiness of machine fault diagnosis by identifying unseen operating domains and reducing unrecognized misdiagnoses caused by changes in fault types, rotational speeds, and noise levels [23]. Song et al. proposed an optimized CNN-BiLSTM framework that combines bidirectional temporal feature learning, improved particle swarm-based hyperparameter optimization, and transfer learning. The method reduces manual parameter-tuning costs and improves bearing fault diagnosis under previously unseen operating conditions with limited labeled samples [24]. Dai et al. proposed a digital twin-assisted graph contrastive domain adaptation framework for small-sample bearing fault diagnosis. By coupling physics-based simulated responses with measured vibration signals and learning domain-invariant representations through graph contrastive adaptation, the method alleviates labeled-data scarcity and distribution discrepancies between digital and physical domains [25].

The belief rule base (BRB) is a representative hybrid knowledge-and-data-driven gray-box modeling approach. It represents expert knowledge through semantically explicit IF–THEN rules, characterizes incomplete, vague, and uncertain information using belief distributions, and aggregates multiple activated rules through the evidential reasoning algorithm. Meanwhile, observational data can be used to optimize model parameters, including rule weights, attribute weights, and belief degrees, thereby balancing diagnostic performance with reasoning transparency. Consequently, BRB has demonstrated considerable potential for modeling small-sample, nonlinear, and uncertain complex systems [26]. It has been widely applied to complex equipment health assessment [27,28], mechanical system fault diagnosis [29,30,31,32], medical decision support [33,34], and student academic performance prediction [35,36], providing an effective framework for uncertainty-aware information fusion and interpretable decision-making across diverse applications.

However, traditional belief rule bases still face two major challenges in bearing fault diagnosis applications. On the one hand, the vibration signal features collected in practice are often not independent of each other. For example, time-domain features such as standard deviation, root mean square, peak-to-peak value, and energy may jointly reflect variations in signal amplitude, resulting in correlations and redundant information among attributes [37]. Directly inputting these correlated attributes into the model may not only cause some information to be repeatedly used during the reasoning process but also increase the model’s sensitivity to noise and operating-condition disturbances, thereby reducing diagnostic accuracy and robustness. On the other hand, traditional belief rule bases generally construct rules based on combinations of multiple attribute referential values. As the number of input attributes or referential levels increases, the number of rules grows exponentially, easily leading to combinatorial rule explosion and consequently increasing the complexity of model construction, optimization, and interpretation.

To address the above challenges, this study aims to develop an interpretable and compact correlation-decoupled interval belief rule base (CD-IBRB) for cross-condition bearing fault diagnosis. The proposed method is designed to reduce redundant diagnostic evidence caused by correlated vibration attributes, avoid the combinatorial growth of conventional BRB rule bases, and improve the stability of fault inference under operating-condition variations and input disturbances. The main contributions of this study are summarized as follows:

1. A correlation-decoupled interval belief rule base (CD-IBRB) is proposed for cross-condition bearing fault diagnosis. Attribute-wise interval rules replace Cartesian-product rule combinations, reducing rule-base complexity while retaining explicit rule representation.
2. A Kendall-rank-correlation-guided attribute decoupling strategy is developed to reduce redundant dependence among selected vibration features and limit repeated evidence propagation during BRB reasoning.
3. P-CMA-ES jointly optimizes belief degrees, rule reliabilities, and rule weights, while evidential reasoning aggregates the activated interval rules.

## 2. BRB Knowledge Base and Problem Description

This section describes the basis of BRB and the problems addressed in this paper.

### 2.1. The Basis of BRB

Belief rule base (BRB) is a typical knowledge–data hybrid modeling method, which can combine expert knowledge and data information to deal with uncertainty in complex system diagnosis. For a fault diagnosis task, the *k*-th belief rule can be expressed as

(1) $$\begin{array}{c} R_{k}:IF\;X_{1}\;is\;A_{1}^{k}\wedge X_{2}\;is\;A_{2}^{k}\wedge \cdots \wedge X_{M}\;is\;A_{M}^{k},\\ THEN\;\{\left(D_{1},\beta_{1,k}\right),\left(D_{2},\beta_{2,k}\right),\ldots ,\left(D_{N},\beta_{N,k}\right)\},\\ with\;rule\;weight\;\theta_{k},attribute\;weight\;\delta_{1},\delta_{1}\cdots ,\delta_{M} \end{array}$$

where $X_{i}$ denotes the *i*-th input attribute, $A_{i}^{k}$ denotes the corresponding reference value or reference interval, $D_{j}$ denotes the *j*-th diagnostic result, and $\beta_{j,k}$ is the belief degree assigned to $D_{j}$ in the *k*-th rule. The rule weight and attribute weight are denoted by $\theta_{k}$ and $\delta_{i}$, respectively.

For cross-condition bearing fault diagnosis, the input sample can be denoted as

(2) $$x=[x_{1},x_{2},\ldots ,x_{M}]$$

and the diagnostic output is

(3) $$y\in \Omega =\{D_{1},D_{2},\ldots ,D_{N}\}$$

The purpose of the model is to construct a reliable mapping relationship between the monitoring attributes and the fault states:

(4) y=f(x,Θ)

where Θ represents the parameter set of the BRB model, including rule weights, attribute weights, rule reliability, and belief degrees. However, when BRB is directly used for cross-condition bearing fault diagnosis, two main problems need to be solved.

### 2.2. Problem Formulation

Problem 1: Attribute correlation in BRB reasoning, In cross-condition bearing fault diagnosis, different vibration features may contain redundant or overlapping information. When correlated attributes are directly used as BRB inputs, the same fault information may be repeatedly activated in the reasoning process, which can reduce the diagnostic accuracy and robustness of the model.

The attribute correlation problem can be formulated as

(5) $$min_{Q}\sum_{i=1}^{M}\sum_{j=i+1}^{M}\left|\tau (x_{i}^{*},x_{j}^{*})\right|$$

where the transformed attribute is defined as

(6) $$x^{*}=xQ^{T}$$

Here, $x=[x_{1},x_{2},\ldots ,x_{M}]$ is the original input vector, $x^{*}=\left[x_{1}^{*},x_{2}^{*},\ldots ,x_{M}^{*}\right]$ is the decoupled input vector, *Q* is the decoupling matrix, and $\tau (x_{i}^{*},x_{j}^{*})$ denotes the Kendall correlation coefficient between the *i*-th and *j*-th decoupled attributes.

To solve this problem, a correlation decoupling strategy is introduced before BRB reasoning. The decoupling matrix *Q* is optimized by minimizing the correlation among transformed attributes. To avoid excessive distortion of the original feature space, an identity constraint is added:

(7) $$min_{Q}\sum_{i=1}^{M}\sum_{j=i+1}^{M}\left|\tau (x_{i}^{*},x_{j}^{*})\right|+\lambda {∥Q-I∥}_{F}^{2}$$

where *I* is the identity matrix, λ is the penalty coefficient, and ${∥\cdot ∥}_{F}$ denotes the Frobenius norm. The transformed attributes $x^{*}$ are then used as the new inputs of the BRB model.

Problem 2: Rule explosion and interval rule construction, Traditional BRB constructs belief rules by combining the reference values of all attributes. When the number of attributes or reference values increases, the number of rules grows rapidly. This rule explosion problem increases computational complexity and weakens the interpretability of the model.

If the *i*-th attribute has $K_{i}$ reference values, the number of traditional BRB rules is

(8) $$R_{\mathrm{BRB}}=\prod_{i=1}^{M}K_{i}$$

where *M* is the number of input attributes. This Cartesian product structure makes the rule base difficult to maintain when *M* or $K_{i}$ is large.

To solve this problem, interval reference values are constructed independently for each attribute. The reference value set of the *i*-th attribute is defined as

(9) $$A_{i}=\left\{A_{i}^{1},A_{i}^{2},\ldots ,A_{i}^{K_{i}}\right\}$$

and adjacent reference values form interval rules:

(10) $$I_{i}^{k}=\left[A_{i}^{k},A_{i}^{k+1}\right],\;k=1,2,\ldots ,K_{i}-1$$

Then, the number of interval rules becomes

(11) $$R_{IBRB}=\sum_{i=1}^{M}\left(K_{i}-1\right)$$

Here, $A_{i}^{k}$ denotes the *k*-th reference value of the *i*-th attribute, $I_{i}^{k}$ denotes the corresponding interval, and $R_{IBRB}$ denotes the number of interval belief rules. Compared with $R_{BRB}$, the interval rule construction reduces the size of the rule base and improves model interpretability while preserving attribute-level diagnostic information.

## 3. Correlation-Aware Decoupling and Robustness Analysis

Attribute correlation has a significant influence on the diagnostic performance and robustness of BRB-based fault diagnosis models. In cross-condition bearing fault diagnosis, different vibration features may contain redundant or overlapping degradation information, which can cause repeated use of correlated evidence during BRB reasoning and weaken the stability of diagnostic results. Therefore, effectively analyzing and reducing attribute correlation is essential for constructing a reliable and interpretable diagnosis model. In this section, a correlation-aware attribute decoupling strategy is developed to transform the original features into a less-correlated representation, and a robustness analysis is introduced to evaluate its effect on improving the stability of the proposed CD-IBRB model.

### 3.1. Correlation-Aware Attribute Transformation

In cross-condition bearing fault diagnosis, several vibration features may describe similar amplitude, fluctuation, or energy information. If these correlated attributes are directly used as BRB inputs, redundant evidence may be repeatedly activated during rule matching and evidence reasoning. Therefore, a correlation-aware attribute transformation is introduced before constructing the interval belief rule base.

Let the original training feature matrix be denoted as

(12) $$X=[x_{1},x_{2},\ldots ,x_{M}]$$

where *M* is the number of input attributes, and $x_{i}$ denotes the sample vector of the *i*-th attribute. To remove the influence of different feature scales, each attribute is standardized by

(13) $$z_{ni}=\frac{x_{ni}-\mu_{i}}{\sigma_{i}}$$

where $x_{ni}$ is the value of the *i*-th attribute in the *n*-th sample, and $\mu_{i}$ and $\sigma_{i}$ are the mean and standard deviation calculated from the training data. The standardized feature matrix is denoted as *Z*.

Kendall rank correlation coefficient is used to measure the dependence between attributes. For the *i*-th and *j*-th attributes, it is defined as

(14) $$\tau_{ij}=\frac{2}{N(N-1)}\sum_{1\leq p<q\leq N}sgn\left(z_{pi}-z_{qi}\right)sgn\left(z_{pj}-z_{qj}\right)$$

where *N* is the number of training samples, and sgn(·) is the sign function. A larger value of $|\tau_{ij}|$ indicates a stronger correlation between two attributes.

To reduce attribute correlation, a decoupling matrix *Q* is introduced to transform the standardized features into a new attribute space:

(15) $$X^{*}=ZQ^{T}$$

where $X^{*}$ denotes the decoupled feature matrix. The matrix *Q* is optimized by minimizing the correlation among transformed attributes while preserving the stability of the original feature space:

(16) $$\begin{array}{c} min_{Q}J\left(Q\right)=\frac{1}{M(M-1)}\sum_{i=1}^{M}\sum_{j=1,j\neq i}^{M}{\left(\tau_{ij}^{*}\right)}^{2}+\\ \lambda_{1}{∥Q-I∥}_{F}^{2}+\lambda_{2}\sum_{i=1}^{M}max{\left(0,\varepsilon_{v}-var\left(x_{i}^{*}\right)\right)}^{2}+\lambda_{3}max(0,\varepsilon_{d}-|det\left(Q\right){\left|\right)}^{2} \end{array}$$

Here, $\tau_{ij}^{*}$ is the Kendall correlation coefficient between the transformed attributes $x_{i}^{*}$ and $x_{j}^{*}$, *I* is the identity matrix, and ${∥\cdot ∥}_{F}$ denotes the Frobenius norm.

In the implementation, the elements of *Q* are bounded by

(17) $$q_{ii}^{min}\leq q_{ii}\leq q_{ii}^{max}$$

(18) $$-q_{ij}^{max}\leq q_{ij}\leq q_{ij}^{max},\;i\neq j$$

where $q_{ii}$ and $q_{ij}$ are the diagonal and non-diagonal elements of *Q*, respectively.

After the optimal decoupling matrix $Q^{*}$ is obtained, each input sample is transformed as

(19) $$x^{*}=\left(\frac{x-\mu }{\sigma }\right){\left(Q^{*}\right)}^{T}$$

The transformed vector $x^{*}$ is then used as the input of the subsequent interval belief rule base.

### 3.2. Robustness Analysis of Attribute Decoupling

In cross-condition bearing fault diagnosis, vibration features often vary with operating conditions, loads, and noise disturbances. Strong correlations among input attributes may cause disturbances in one attribute to propagate to other correlated attributes. Consequently, correlated information may be repeatedly used during BRB reasoning, thereby reducing the stability of diagnostic results. Therefore, the robustness of the attribute decoupling strategy should be evaluated before constructing the CD-IBRB mode.

For an input sample *x*, the disturbed sample is defined as

$$x^{\prime }=x+\Delta x$$

where Δx denotes the external disturbance or feature fluctuation. After standardization and attribute decoupling, the original sample and the disturbed sample are transformed as

(20) $$x^{*}=\left(\frac{x-\mu }{\sigma }\right)Q^{T}$$

(21) $$x^{\prime *}=\left(\frac{x^{\prime }-\mu }{\sigma }\right)Q^{T}$$

where μ and σ are the mean and standard deviation obtained from the training data, and *Q* is the optimized decoupling matrix. The disturbance after decoupling can be expressed as

(22) $$\Delta x^{*}=x^{\prime *}-x^{*}=\left(\frac{\Delta x}{\sigma }\right)Q^{T}$$

This equation shows that the influence of input disturbance on the transformed attributes is controlled by the decoupling matrix. By reducing the correlation among transformed attributes, the disturbance is less likely to be repeatedly amplified through correlated attributes in the subsequent rule activation process.

To evaluate the robustness of attribute decoupling, the change in the output belief distribution before and after disturbance is measured. Let the output belief distribution of the CD-IBRB model be

(23) $$\beta \left(x^{*}\right)=\left[\beta_{1}\left(x^{*}\right),\beta_{2}\left(x^{*}\right),\ldots ,\beta_{C}\left(x^{*}\right)\right]$$

where *C* is the number of fault classes, and $\beta_{c}\left(x^{*}\right)$ is the belief degree assigned to the *c*-th fault class. After disturbance, the output belief distribution becomes $\beta (x^{\prime *})$. The robustness measure can be defined as

(24) $$R_{\beta }=\frac{1}{S}\sum_{n=1}^{S}{∥\beta \left(x_{n}^{*}\right)-\beta \left(x_{n}^{\prime *}\right)∥}_{2}$$

where *S* is the number of test samples, and ${∥\cdot ∥}_{2}$ denotes the Euclidean norm. A smaller $R_{\beta }$ indicates that the model output changes less under feature disturbance, which means better robustness.

In addition, the robustness of the diagnostic decision can be evaluated by the accuracy retention rate under disturbance:

(25) $$R_{acc}=\frac{Acc_{\eta }}{Acc_{0}}$$

where $Acc_{0}$ is the diagnostic accuracy without disturbance, and $Acc_{\eta }$ is the diagnostic accuracy when the disturbance level is η. A larger $R_{acc}$ indicates that the model can maintain more stable diagnostic performance under disturbed or cross-condition samples.

Therefore, the robustness analysis of attribute decoupling contains two aspects. First, the decoupling matrix reduces the correlation among input attributes and weakens the propagation of redundant disturbances. Second, the stability of the CD-IBRB model can be evaluated by the variation of output belief distributions and the accuracy retention rate under different disturbance levels. In the cross-condition bearing fault diagnosis experiment, these measures are used to analyze whether the proposed attribute decoupling strategy improves the stability and reliability of the diagnosis model.

## 4. Construction of the CD-IBRB Fault Diagnosis Model

**Figure 1:**
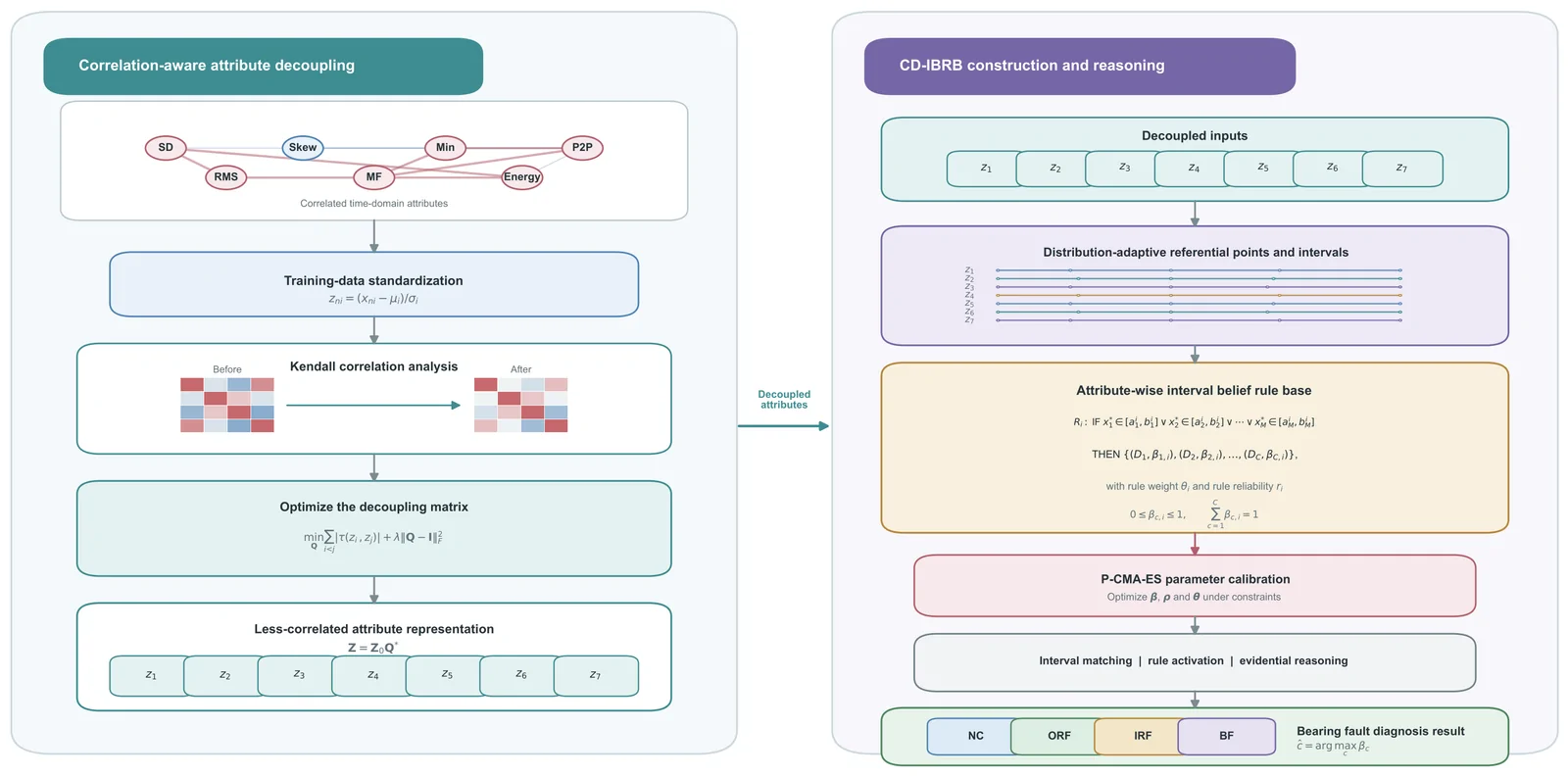
Correlation decoupling and diagnostic framework of the proposed CD-IBRB model.

This section presents the complete modeling framework of the proposed CD-IBRB model, whose overall architecture is illustrated in Figure 1. Section 4.1 describes the acquisition of input attributes, including time-domain feature extraction and XGBoost-based feature importance analysis. Section 4.2 details the construction of the CD-IBRB model, including attribute correlation decoupling and the generation of referential points, referential intervals, and interval-based belief tables. Section 4.3 introduces the reasoning mechanism, covering input transformation, rule activation, and evidential reasoning aggregation. Section 4.4 presents the parameter optimization strategy for jointly adjusting the belief degrees, rule reliabilities, and rule weights. Finally, Section 4.5 evaluates the robustness of attribute decoupling under input disturbances and cross-condition scenarios.

### 4.1. Input Attribute Extraction and Selection

To obtain informative and interpretable inputs for the CD-IBRB model, bearing vibration signals are first collected using acceleration sensors, from which representative time-domain features are extracted. XGBoost-based feature importance analysis is then employed to identify diagnostically relevant attributes and eliminate redundant or noise-sensitive information. This process reduces the influence of irrelevant features while retaining the physical information contained in the vibration signals. The time-domain feature extraction and attribute importance analysis procedures are presented in Section 4.1.1 and Section 4.1.2, respectively.

#### 4.1.1. Time-Domain Feature Extraction

Time-domain feature analysis was first performed on each segmented bearing vibration signal to extract diagnostically relevant information from its amplitude variation and statistical distribution. Specifically, 13 candidate features were calculated, including the mean, standard deviation (SD), skewness, kurtosis, maximum, minimum, peak-to-peak value (P2P), root mean square (RMS), crest factor, shape factor, impulse factor, margin factor, and energy. These features characterize the vibration signals from complementary perspectives, including central tendency, dispersion, distribution asymmetry, impulsiveness, extreme amplitude, and overall signal energy. Each signal segment was therefore transformed into a 13-dimensional candidate attribute vector, providing physically interpretable inputs for subsequent feature importance analysis. The definitions and calculation procedures of these time-domain features followed those reported in Ref. [3], and all feature extraction methods were implemented consistently with that study. This process retained the principal statistical and physical information contained in the original vibration signals while providing a structured attribute representation for the CD-IBRB model.

#### 4.1.2. XGBoost-Based Attribute Importance Evaluation

Following time-domain feature extraction, each vibration sample was represented by 13 candidate attributes. The resulting feature matrix can be expressed as $X=\left[x_{1},x_{2},\ldots ,x_{13}\right]\in R^{N_{s}\times 13},$where $N_{s}$ denotes the number of samples and $x_{j}$ represents the sample vector associated with the *j*-th time-domain attribute. Although these attributes describe the vibration signals from different statistical perspectives, their contributions to fault identification are not identical. Some attributes may contain limited discriminative information or exhibit redundant responses to changes in bearing condition. Directly using all candidate attributes would therefore increase the input dimension and subsequent rule-base complexity.

To identify the attributes most relevant to bearing fault diagnosis, XGBoost was employed to evaluate feature importance. By iteratively constructing boosted decision trees, XGBoost can capture nonlinear relationships between the candidate attributes and fault categories. The contribution of each attribute to the tree-based classification process was accumulated to obtain its importance score.

Let $I_{j}$ denote the original importance score of the *j*-th attribute. Its normalized importance score, denoted by $S_{j}$, is calculated as

(26) $$S_{j}=\frac{I_{j}}{\sum_{k=1}^{1}3I_{k}},\;j=1,2,\ldots ,13.$$

**Figure 2:**
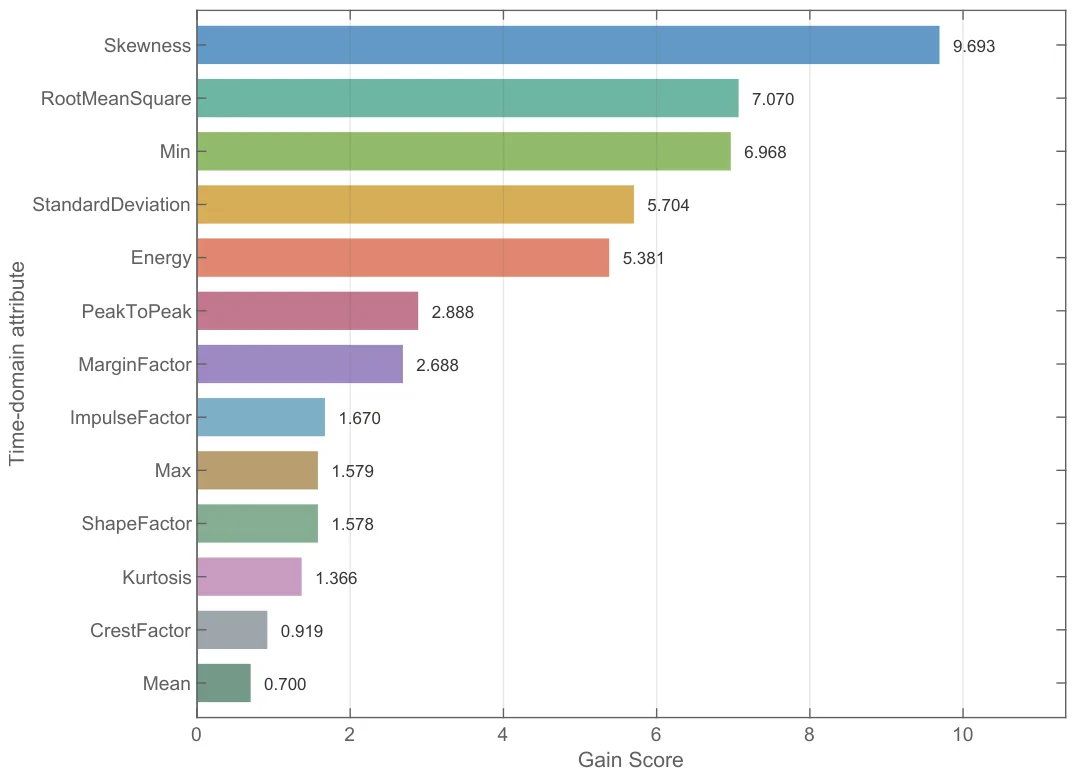
Attribute importance ranking based on XGBoost.

A larger $S_{j}$ indicates that the corresponding attribute contributes more strongly to bearing fault identification. The attributes were ranked in descending order according to $S_{j}$, and the resulting feature importance ranking is presented in Figure 2.

Based on this ranking, seven attributes with relatively high diagnostic contributions were retained: standard deviation (SD), skewness, minimum, peak-to-peak value (P2P), root mean square (RMS), margin factor, and energy. These attributes characterize amplitude dispersion, distribution asymmetry, extreme values, impulsive behavior, and signal energy. Consequently, the original 13-dimensional feature space was reduced to a seven-dimensional attribute space. The selected attributes were subsequently used as inputs to the correlation-aware attribute transformation, thereby reducing redundant information and the complexity of the CD-IBRB model while preserving physically interpretable fault characteristics.

### 4.2. Construction and Initialization of the Interval Belief Rule Base

The CD-IBRB model is constructed based on IF–THEN belief rules, and each rule is represented by an interval form. After correlation-aware attribute transformation, the decoupled attributes are used as the inputs of the interval rule base. Assuming that the decoupled input attributes are conditionally independent in the reasoning process, the relationship between the input intervals and the fault diagnosis results can be described as follows.

(27) $$\begin{array}{c} R_{l}:\;IF\;x_{1}^{*}\in \left[a_{1}^{l},b_{1}^{l}\right]\vee x_{2}^{*}\in \left[a_{2}^{l},b_{2}^{l}\right]\vee \cdots \vee x_{M}^{*}\in \left[a_{M}^{l},b_{M}^{l}\right]\\ THEN\;\{\left(D_{1},\beta_{1,l}\right),\left(D_{2},\beta_{2,l}\right),\ldots ,\left(D_{C},\beta_{C,l}\right)\},\;\\ with\;rule\;weight\;\theta_{l}\;and\;rule\;reliability\;r_{l}\\ 0\leq \beta_{c,l}\leq 1,\;\sum_{c=1}^{C}\beta_{c,l}=1 \end{array}$$

where $R_{l}$ denotes the *l*-th belief rule in the CD-IBRB model. $x_{i}^{*}$ is the *i*-th decoupled input attribute, and $I_{i}^{k}$ denotes the *k*-th referential interval of the *i*-th attribute. $D_{c}$ represents the *c*-th fault diagnosis level, and $\beta_{c,l}$ is the belief degree assigned to $D_{c}$ in the *l*-th rule. $\theta_{l}$ and $r_{l}$ denote the rule weight and rule reliability of the *l*-th rule, respectively. *C* is the number of fault classes. The belief degrees satisfy the normalization constraint, which ensures that each rule provides a complete belief distribution over all fault classes.

#### 4.2.1. Construction of Referential Points and Intervals

After the correlation-aware attribute transformation, the decoupled attributes are used to construct the interval belief rule base. The construction process consists of four steps. First, the distribution characteristics of each decoupled attribute are analyzed using skewness and kurtosis. Second, the number of referential points is determined by considering distribution resolution, sample support within individual intervals, and rule-base complexity. Third, the referential points are generated according to the statistical distribution of each attribute. Finally, adjacent referential points are connected to form referential intervals, with each interval corresponding to an attribute-wise belief rule. Unlike conventional BRB models that construct rules through the Cartesian product of multiple referential values, CD-IBRB constructs interval rules independently for individual attributes. This attribute-wise structure reduces the number of rules while retaining a transparent relationship between the input intervals and diagnostic results.

Let the decoupled training feature matrix be denoted as

(28) $$X^{*}=\left[x_{1}^{*},x_{2}^{*},\ldots ,x_{M}^{*}\right]$$

where *M* is the number of input attributes, and $x_{i}^{*}$ represents the sample vector of the *i*-th decoupled attribute. For the *i*-th attribute, the referential point set is defined as

(29) $$A_{i}=\left\{A_{i}^{1},A_{i}^{2},\ldots ,A_{i}^{K_{i}}\right\}$$

where $K_{i}$ is the number of referential points assigned to the *i*-th attribute. To ensure the interpretability of interval division, the referential points should satisfy

(30) $$A_{i}^{1}<A_{i}^{2}<\cdots <A_{i}^{K_{i}}$$

The number of referential points is treated as a structural parameter of the CD-IBRB model and is determined before parameter optimization. Its selection requires a balance among distribution resolution, sample support within individual intervals, and rule-base complexity. Too few referential points may produce excessively broad intervals and obscure local differences among fault classes. Conversely, too many referential points may reduce the number of training samples covered by each interval, resulting in unstable estimates of the initial belief distributions and rule reliabilities while increasing the number of parameters to be optimized. In the present bearing fault diagnosis experiment, 11 referential points are assigned to each of the seven decoupled attributes, producing ten intervals per attribute and 70 interval rules in total. This setting provides sufficient granularity to characterize local variations in the attribute distributions while maintaining adequate sample support and a compact rule structure. The number of referential points is determined exclusively from the characteristics of the training dataset and remains fixed during model optimization and testing.

The distribution characteristics of each decoupled attribute are subsequently evaluated. The skewness and kurtosis of the *i*-th attribute are calculated as

(31) $$S_{i}=\frac{E[{\left(x_{i}^{*}-\mu_{i}^{*}\right)}^{3}]}{{\left(\sigma_{i}^{*}\right)}^{3}}$$

(32) $$K_{i}^{u}=\frac{E[{\left(x_{i}^{*}-\mu_{i}^{*}\right)}^{4}]}{{\left(\sigma_{i}^{*}\right)}^{4}}$$

where $\mu_{i}^{*}$ and $\sigma_{i}^{*}$ are the mean and standard deviation of the *i*-th decoupled attribute, respectively. An attribute is regarded as having a normal-like distribution when the absolute skewness does not exceed 1 and the absolute difference between its kurtosis and 3 does not exceed 1. These thresholds provide an explicit criterion for identifying attributes whose samples are mainly concentrated around the mean without excessive asymmetry or heavy-tailed behavior.

For a normal-like attribute, the referential points are generated around the mean using equal spacing:

(33) $$A_{i}^{k}=\mu_{i}^{*}+d_{i}^{k}\Delta_{i},\;k=1,2,\ldots ,K_{i}$$

where $d_{i}^{k}$ denotes the relative position of the *k*-th referential point and $\Delta_{i}$ denotes the interval step of the *i*-th attribute. An attribute is classified as normal-like when its absolute skewness does not exceed 1 and the absolute difference between its kurtosis and 3 does not exceed 1. For the 11-point setting, the relative positions are fixed from −4.5 to 5.5 with an increment of 1. The interval step $\Delta_{i}$ is determined as the largest of three candidate values: the value required to extend the lower boundary to three standard deviations below the mean, the distance from the mean to the observed maximum divided by 5.5, and the distance from the observed minimum to the mean divided by 4.5. This rule ensures that the resulting referential points cover both the principal statistical range and the complete observed range of the training attribute.

Adjacent referential points are sequentially connected to form ten mutually exclusive intervals. The first nine intervals are left-closed and right-open, whereas the final interval includes both boundaries. During inference, a test value below the first referential point is assigned to the first interval, while a value above the final referential point is assigned to the last interval. All distribution statistics, referential points, interval steps, and interval boundaries are calculated exclusively from the training set and remain fixed during testing to prevent data leakage.

#### 4.2.2. Belief Table Generation Based on Interval Rules

Unlike the traditional BRB rule combination method, the CD-IBRB model constructs the belief table through interval addition. When at least one decoupled attribute $x_{i}^{*}$ falls within the corresponding referential interval $a_{i}^{l},b_{i}^{l}$, the interval rule $R_{l}$ is activated and incorporated into the belief table. The interval belief rule is expressed as

(34) $$\begin{array}{c} R_{l}:\;IF\;x_{1}^{*}\in \left[a_{1}^{l},b_{1}^{l}\right]\vee x_{2}^{*}\in \left[a_{2}^{l},b_{2}^{l}\right]\vee \cdots \vee x_{M}^{*}\in \left[a_{M}^{l},b_{M}^{l}\right],\\ THEN\;\left\{\begin{array}{c} \left(D_{1},\beta_{1,l}\right),\left(D_{2},\beta_{2,l}\right),\ldots ,\left(D_{C},\beta_{C,l}\right) \end{array}\right\}. \end{array}$$

The initial belief degree $\beta_{c,l}^{0}$ is determined according to the class distribution of the training samples associated with rule $R_{l}$:

(35) $$\beta_{c,l}^{0}=\frac{N_{c,l}+\eta }{\sum_{d=1}^{C}N_{d,l}+C\eta }$$

where $N_{c,l}$ denotes the number of training samples belonging to class $D_{c}$ and satisfying the antecedent intervals of rule $R_{l}$, and η is the smoothing coefficient. In this way, the interval rules and their initial belief distributions jointly form the belief table of the CD-IBRB model.

### 4.3. Reasoning Mechanism of the CD-IBRB Model

#### 4.3.1. Input Information Transformation

For a test sample, the decoupled input vector is denoted as $x^{*}=\left[x_{1}^{*},x_{2}^{*},\ldots ,x_{M}^{*}\right]$. For rule $R_{l}$, the matching degree between the *i*-th input attribute and its corresponding referential interval is defined as

(36) $$\alpha_{i}^{l}=\left\{\begin{array}{cc} 1, & x_{i}^{*}\in \left[a_{i}^{l},b_{i}^{l}\right],\\ 0, & x_{i}^{*}\notin \left[a_{i}^{l},b_{i}^{l}\right], \end{array}\right.\;i=1,\ldots ,M,\;l=1,\ldots ,L.$$

Because the antecedent attributes in Equation (27) are connected by the logical operator ∨, the overall matching degree of rule $R_{l}$ is calculated as $\alpha_{l}=max_{1\leq i\leq M}\alpha_{i}^{l}$. Accordingly, rule $R_{l}$ is activated when $\alpha_{l}>0$. The set of activated rules is expressed as

(37) $$R^{a}\left(x^{*}\right)=\left\{\begin{array}{c} R_{l}|\alpha_{l}>0,\;l=1,\ldots ,L \end{array}\right\}$$

where $\alpha_{i}^{l}$ denotes the interval matching degree of the *i*-th attribute in rule $R_{l}$, $\alpha_{l}$ denotes the overall matching degree of rule $R_{l}$, and $R^{a}\left(x^{*}\right)$ is the activated rule set.

#### 4.3.2. Calculation of Rule Activation Weights

Let $\delta_{i}$ denote the relative weight of the *i*-th input attribute, and let ${\bar{\delta }}_{i}$ denote its normalized value. After the attribute matching degrees have been obtained, the activation weight of rule $R_{l}$ is calculated as

(38) $$\omega_{l}=\frac{\theta_{l}r_{l}\prod_{i=1}^{M}{\left(\alpha_{i}^{l}\right)}^{{\bar{\delta }}_{i}}}{\sum_{q=1}^{L}\left[\theta_{q}r_{q}\prod_{i=1}^{M}{\left(\alpha_{i}^{q}\right)}^{{\bar{\delta }}_{i}}\right]},\;{\bar{\delta }}_{i}=\frac{\delta_{i}}{\max_{1\leq j\leq M}\left\{\delta_{j}\right\}}$$

where $\omega_{l}$ denotes the normalized activation weight of rule $R_{l}$, $\theta_{l}$ and $r_{l}$ denote its rule weight and rule reliability, respectively, and $\alpha_{i}^{l}$ represents the matching degree between the *i*-th input attribute and its antecedent interval in rule $R_{l}$. The rule reliability explicitly regulates the contribution of each rule to the reasoning process. A rule with a larger $r_{l}$ provides stronger support for the final result, whereas a rule with $r_{l}=0$ does not participate in the subsequent ER aggregation. Rule $R_{l}$ is activated when $\omega_{l}>0$.

#### 4.3.3. Aggregation of Activated Rules

After the normalized activation weights have been obtained, the evidential reasoning (ER) algorithm is employed to aggregate the consequent belief distributions of the activated rules [38]. The fused belief degree associated with fault class $D_{c}$ is calculated as

(39) $${\hat{\beta }}_{c}=\frac{\mu \left[\prod_{l=1}^{L}\left(\omega_{l}\beta_{c,l}+1-\omega_{l}\sum_{d=1}^{C}\beta_{d,l}\right)-\prod_{l=1}^{L}\left(1-\omega_{l}\sum_{d=1}^{C}\beta_{d,l}\right)\right]}{1-\mu \prod_{l=1}^{L}\left(1-\omega_{l}\right)}$$

The normalization factor μ is calculated as

(40) $$\mu ={\left[\sum_{c=1}^{C}\prod_{l=1}^{L}\left(\omega_{l}\beta_{c,l}+1-\omega_{l}\sum_{d=1}^{C}\beta_{d,l}\right)-\prod_{l=1}^{L}\left(1-\omega_{l}\sum_{d=1}^{C}\beta_{d,l}\right)\right]}^{-1}$$

where $\hat{\beta }c$ denotes the fused belief degree associated with fault class $D_{c}$, βc,l is the belief degree assigned to $D_{c}$ by rule $R_{l}$, *L* is the total number of rules, and *C* is the number of fault classes. Since the rule reliability $r_{l}$ is explicitly incorporated into the activation weight $\omega_{l}$ in Equation (38), its influence is propagated through the ER aggregation in Equations (39) and (40).

The fault class corresponding to the maximum fused belief degree is selected as the final diagnostic result: $\hat{y}=max_{c\in \{1,\ldots ,C\}}{\hat{\beta }}_{c}$.

### 4.4. Parameter Optimization of the CD-IBRB Model

The initial belief degrees, rule reliabilities, and rule weights of the CD-IBRB model are determined from the training samples, expert knowledge, and prior parameter settings. However, the limited availability of training samples and the subjectivity of expert judgments may prevent these initial parameters from accurately representing the nonlinear relationship between the input attributes and fault classes. Therefore, the projection covariance matrix adaptation evolution strategy (P-CMA-ES) is employed to jointly optimize the consequent belief degrees $\beta_{c,l}$, rule weights $\theta_{l}$, and rule reliabilities $r_{l}$. As a derivative-free evolutionary algorithm, P-CMA-ES adaptively updates the search covariance matrix and step size to capture dependencies among coupled parameters and explore complex nonconvex solution spaces. Meanwhile, its projection mechanism maps candidate solutions onto the feasible region, ensuring that the normalization and equality constraints of the belief parameters are satisfied [39]. Consequently, P-CMA-ES can improve diagnostic performance while preserving the explicit rule structure and interpretability of CD-IBRB.

To improve the diagnostic performance of the CD-IBRB model, cross-entropy loss is adopted as the objective function. The belief degrees, rule weights, and rule reliabilities are jointly optimized as follows:

(41) $$D_{best}=optimize\;\left(\beta_{c,l},\theta_{l},r_{i}\right)=min\;CE\left(\beta_{c,l},\theta_{l},r_{i}\right)$$

where $D_{best}$ denotes the best feasible parameter solution obtained by P-CMA-ES. The cross-entropy loss $CE(\beta_{c,l},\theta_{l},r_{i})$ is calculated as

(42) $$CE\left(\beta_{c,l},\theta_{l},r_{i}\right)=-\frac{1}{p}\sum_{p=1}^{P}\sum_{n=1}^{N}y_{p,c}ln{\hat{\beta }}_{p,c}\left(\beta_{c,l},\theta_{l},r_{l}\right)$$

where *P* denotes the number of training samples and *C* is the number of fault classes. The variable $y_{p,c}$ is the true class indicator of the *p*-th training sample, such that $y_{p,c}=1$ if sample *p* belongs to fault class $D_{c}$; otherwise, $y_{p,c}=0$. The term ${\hat{\beta }}_{p,c}$ denotes the final fused belief degree assigned to fault class $D_{c}$ by the CD-IBRB model after the ER aggregation in Equation (39).

The parameter optimization problem is subject to the following boundary and normalization constraints:

(43) $$\begin{array}{c} minCE(\beta_{c,l},\theta_{l},r_{i})\\ s.t.\;0\leq \theta_{l}\leq 1,l=1,\ldots ,L,\\ 0\leq r_{l}\leq 1,l=1,\ldots ,L\\ 0\leq \beta_{c,l}\leq 1,c=1,\ldots ,C,l=1,\ldots ,L\\ \sum_{c=1}^{C}\beta_{c,l}=1,l=1,\ldots ,L \end{array}$$

The first two constraints ensure that the rule weights and rule reliabilities remain within their valid ranges. The remaining constraints ensure that the consequent belief degrees are nonnegative and form a complete belief distribution for each interval rule. Under these constraints, P-CMA-ES searches for the parameter solution that minimizes the cross-entropy loss while preserving the validity of the CD-IBRB rule base.

The steps of the P-CMA-ES are as follows:

Step 1: Initial operation

The P-CMA-ES algorithm is initialized by specifying the initial mean vector $mean^{0}=\Omega^{0}$ and the initial optimization vector $\Omega^{0}$. The initial covariance matrix $C^{0}$, step size $\varepsilon_{0}$, population size λ, and number of selected offspring τ are then configured to guide the evolutionary search.

Step 2: Sampling operation

Equation (44) is used to generate the offspring population during each sampling iteration:

(44) $$\Omega_{k}^{\left(g+1\right)}=mean^{\left(g\right)}+\varepsilon^{\left(g\right)}N\left(0,C^{\left(g\right)}\right),k=1,2,\ldots ,\lambda$$

where $\Omega_{k}^{\left(g+1\right)}$ denotes the *i*-th candidate solution in generation *(g + 1)*th, $mean^{\left(g\right)}$ is the mean vector of generation *g*th, and $\varepsilon^{\left(g\right)}$ represents the corresponding step size. In addition, N is a multivariate normal distribution with zero mean and covariance matrix $C^{\left(g\right)}$.

Step 3: Projection operation

The projection operation is formulated as Equation (45):

(45) $$\begin{array}{c} \Omega_{k}^{\left(g+1\right)}\left(1+n_{e}\times \left(j-1\right):n_{e}\times j\right)=\Omega_{k}^{\left(g+1\right)}(1+n_{e}\times \left(j-1\right)\\ :n_{e}\times j)-A_{e}^{T}\times {\left(A_{e}\times A_{e}^{T}\right)}^{-1}\times \Omega_{k}^{\left(g+1\right)}(1+n_{e}\times \left(j-1\right)\\ :n_{e}\times j)\times A_{e} \end{array}$$

To satisfy the parameter constraints, the equality conditions are expressed as hyperplane equations and enforced through a projection operation, as shown in Formula (46):

(46) $$A_{e}\Omega_{k}^{\left(g+1\right)}\left(1+n_{e}\times \left(j-1\right):n_{e}\times j\right)=1,j=1,2,\ldots ,N+1$$

where $n_{e}$ denotes the number of variables subject to equality constraints in the solutions $\Omega_{k}^{\left(g+1\right)}$, and j=1,2,…,N+1. $A_{e}={\left[1\ldots 1\right]}_{1\times N}$ represents the corresponding candidate parameter vector.

Step 4: Select operation

The top-performing candidate solutions are selected according to Equation (47) and used to update the population mean.

(47) $$mean^{\left(g+1\right)}=\sum_{i=1}^{\tau }\omega_{i}\Omega_{i:\lambda }^{\left(g+1\right)}$$

where $\Omega_{i:\lambda }^{\left(g+1\right)}$ denotes the *i*-th ranked candidate among the λ offspring solutions in generation *g*th, and τ is the offspring population size.

Step 5: Adapting operation

The covariance matrix is adaptively updated throughout the evolutionary search. This process is repeated until the optimal parameter vector $\Omega_{optimal}$ is obtained, which is then used to construct the optimized CD-IBRB model. The covariance matrix is updated according to Equations (48) and (49), whereas the step size σ is adjusted using Equation (50).

(48) $$\begin{array}{c} C^{\left(g+1\right)}=\left(1-c_{1}-c_{2}\right)•C^{\left(g\right)}+c_{1}p_{c}^{\left(g+1\right)}{\left(p_{c}^{\left(g+1\right)}\right)}^{T}\\ +c_{2}\sum_{i=1}^{\tau }\omega_{i}\left(\frac{\Omega_{i:\lambda }^{\left(g+1\right)}-mean^{\left(g\right)}}{\varepsilon^{\left(g\right)}}\right){\left(\frac{\Omega_{i:\lambda }^{\left(g+1\right)}-mean^{\left(g\right)}}{\varepsilon^{\left(g\right)}}\right)}^{T} \end{array}$$

(49) $$\begin{array}{c} p_{c}^{\left(g+1\right)}=\left(1-c_{c}\right)•p_{c}^{\left(g\right)}+\sqrt{c_{c}\left(2-c_{c}\right)}•{\left(\sum_{i=1}^{\tau }w_{i}^{2}\right)}^{-0.5}\\ •(mean^{\left(g+1\right)}-mean^{\left(g\right)})/\varepsilon^{\left(g\right)} \end{array}$$

(50) $$\varepsilon^{g+1}=\varepsilon^{g}\exp\left(\frac{c_{\sigma }}{d_{\sigma }}\left(\frac{∥p_{\sigma }^{g+1}∥}{E∥N(0,1)∥}-1\right)\right)$$

### 4.5. Robustness Evaluation of Attribute Decoupling

Attribute correlation may amplify the influence of input disturbances and reduce the stability of diagnostic results. Following the robustness analysis in the correlation-decoupling method, the Lipschitz index is employed to quantify the sensitivity of the CD-IBRB model to input perturbations.

For an input sample *x*, a perturbed sample is generated as

(51) $$x^{\prime }=x+\Delta x$$

where Δx denotes a small random disturbance. After attribute decoupling, the corresponding inputs are $x^{*}$ and $x^{\prime *}$. Let $B(x^{*})$ and $B(x^{\prime *})$ denote the fused belief distributions produced by the CD-IBRB model. The local Lipschitz index is defined as

(52) $$\varpi \left(x\right)=\frac{{∥B\left(x^{*}\right)-B\left(x^{\prime *}\right)∥}_{1}}{{∥x^{*}-x^{\prime *}∥}_{1}}$$

The robustness of the model is evaluated by the maximum Lipschitz index over the test samples:

(53) $$LIP=max_{n=1,2,\ldots ,N_{s}}\varpi \left(x_{n}\right)$$

where $N_{s}$ is the number of test samples. A smaller LIP indicates that the output belief distribution changes less under input disturbances, demonstrating stronger robustness. The robustness of attribute decoupling is verified by comparing the LIP values before and after the decoupling operation.

## 5. Case Study

To evaluate the effectiveness and generalizability of the proposed CD-IBRB method, two case studies were conducted. First, CD-IBRB was applied to a cross-condition bearing fault diagnosis task using an engineering-oriented bearing dataset to evaluate its diagnostic accuracy, interpretability, and robustness under varying operating conditions. Second, five public classification datasets from the UCI and KEEL repositories were employed to assess the generalization capability of the proposed method across datasets with different attribute structures and class distributions. Together, these case studies provide a systematic evaluation of the diagnostic performance, adaptability, and stability of CD-IBRB under diverse application scenarios.

### 5.1. Background Description

Bearings are critical components of rotating machinery and are extensively used in aero-engines, wind turbines, steam turbines, compressors, and other large-scale equipment. Their health condition directly affects the operational safety and reliability of the entire mechanical system. Under high-speed, heavy-load, and variable-condition environments, bearing components, including the rolling elements, inner race, outer race, and cage, are susceptible to fatigue spalling, wear, and other forms of damage. If these faults are not identified promptly, they may develop into severe equipment failures or even safety accidents. Therefore, accurate bearing fault diagnosis is essential for ensuring reliable equipment operation and supporting condition-based maintenance. In this study, a rolling-bearing dataset provided by Shandong University of Science and Technology was employed to evaluate the fault diagnosis performance of the proposed CD-IBRB model.

The dataset was collected using the bearing fault simulation platform developed by Shandong University of Science and Technology (https://github.com/JRWang-SDUST/SDUST-Dataset.git, accessed on 18 August 2026). The experimental platform mainly consisted of an AC motor, a motor speed controller, a test bearing, a planetary gearbox, a load-adjustment handle, and a magnetic powder brake. Two three-axis piezoelectric accelerometers were magnetically mounted on the bearing housing and the fixed brackets at both ends of the planetary gearbox to measure vibration signals from the bearing and gearbox, respectively. Signal acquisition was performed using the LMS Test, a lab vibration and noise measurement system. For each acquisition, vibration signals were recorded for 40 s at a sampling frequency of 25.6 kHz and stored as six-channel .mat files according to the corresponding fault type and operating condition. The dataset covered both constant and varying operating conditions. Under constant conditions, the rotational speeds were set to 1000, 1500, 1800, 2000, 2500, and 3000 r/min, while the radial loads were set to 0, 20, 40, and 60 N. Under varying-speed conditions, three speed ranges were considered, namely 800–1500, 1000–2000, and 1500–2500 r/min, with the same four load levels.

**Figure 3:**
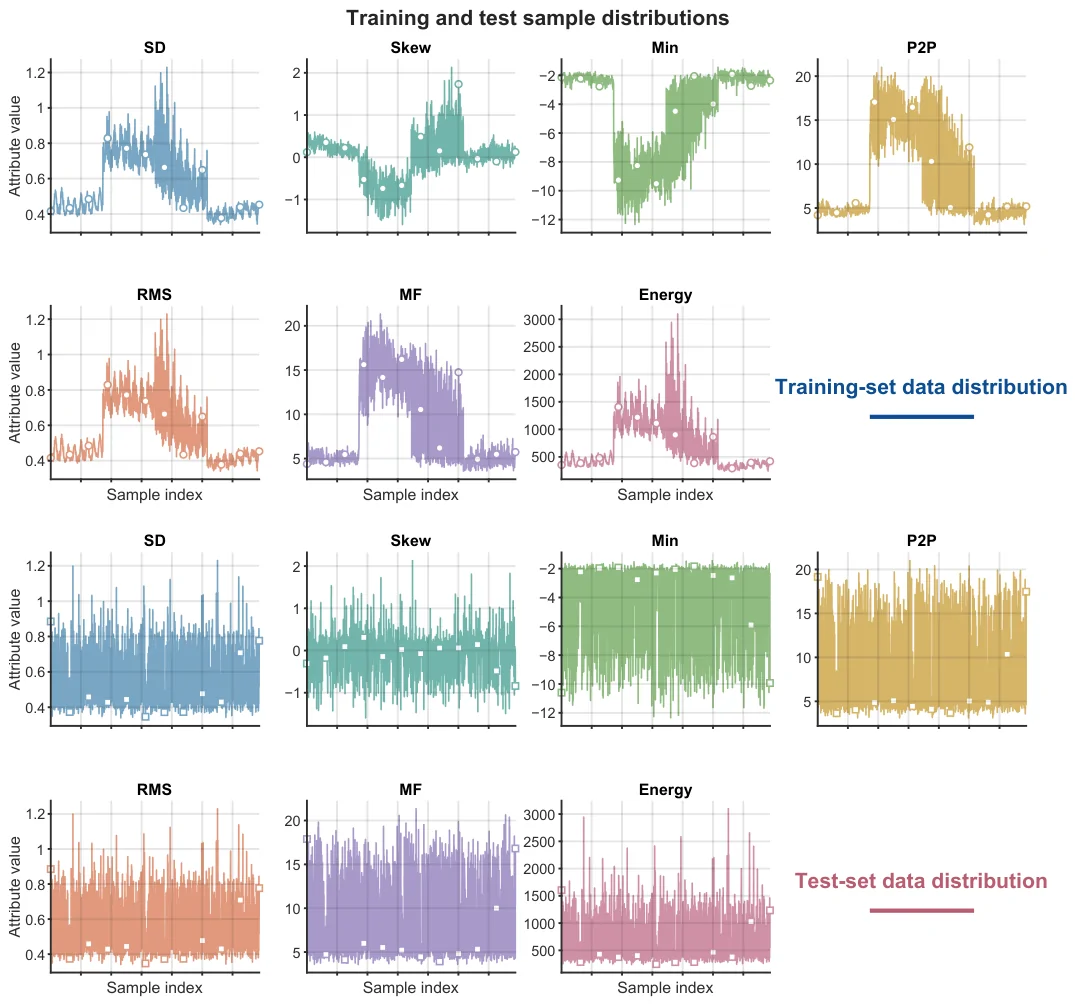
Distribution of training and test samples in the experimental dataset.

Bearing defects of different severities were introduced into the inner race, outer race, and rolling elements using wire-cut electrical discharge machining. The defect widths were set to 0.2, 0.4, and 0.6 mm. Accordingly, the collected signals covered four health conditions: normal condition (NC), inner-race fault (IRF), outer-race fault (ORF), and ball fault (BF), with each fault type containing three defect severities. For the cross-condition diagnosis experiment, signals corresponding to a defect width of 0.2 mm and a rotational speed of 2000 r/min were selected under radial loads of 0 and 20 N. The first vibration channel was selected for subsequent analysis. To prevent data leakage, samples collected under the two load conditions were maintained as strictly separate operating-condition subsets. The continuous vibration signals were segmented using a window length of 2048 data points and a step size of 3048 data points. Because the step size exceeded the window length, adjacent segments contained no shared signal points and were separated by a 1000-point interval, thereby eliminating sample overlap and reducing dependence between neighboring observations. The sample distribution is illustrated in Figure 3, and detailed information on the experimental dataset is provided in Table 1.

The present study evaluates CD-IBRB as an offline diagnostic decision-support method rather than as a certified industrial condition-monitoring system. Its vibration-monitoring and data-processing workflow was designed with reference to the general principles of ISO 13373-1:2002 [40] and ISO 13373-2:2016 [41], while the interpretation of diagnostic information is conceptually consistent with ISO 13379-1:2025 [42]. The terminology concerning rolling-bearing damage and failure is referenced against ISO 15243:2017 [43]. Nevertheless, formal industrial deployment would require site-specific verification of sensor installation, measurement quality, operating-condition coverage, alarm thresholds, and maintenance criteria.

### 5.2. Construction of the CD-IBRB Model for Bearing Fault Diagnosis

This section presents the construction and parameter configuration of the CD-IBRB model for cross-condition bearing fault diagnosis. First, the input attributes are selected based on their importance and transformed through attribute decoupling. Next, attribute-specific referential points and intervals are determined, and the corresponding belief table is initialized using the training samples. Finally, the belief degrees, rule reliabilities, and rule weights are optimized using P-CMA-ES to obtain the final CD-IBRB diagnosis model.

Step 1: Acquisition of Input Attributes

Following the procedure described in Section 4.1, 13 time-domain features are initially extracted from each vibration signal segment. Based on the XGBoost feature-importance ranking, seven diagnostically relevant attributes are retained: standard deviation (SD), skewness, minimum value (Min), peak-to-peak value (P2P), root mean square (RMS), margin factor (MF), and energy. The selected input vector is expressed as

(54) $$x=\left[x_{1},x_{2},\ldots ,x_{7}\right]=\left[SD,Skewness,Min,P2P,RMS,MF,Energy\right]$$

These attributes are subsequently standardized and transformed using the attribute decoupling method. The resulting decoupled attributes are used as the final inputs of the CD-IBRB model.

Step 2: Attribute Decoupling

**Figure 4:**
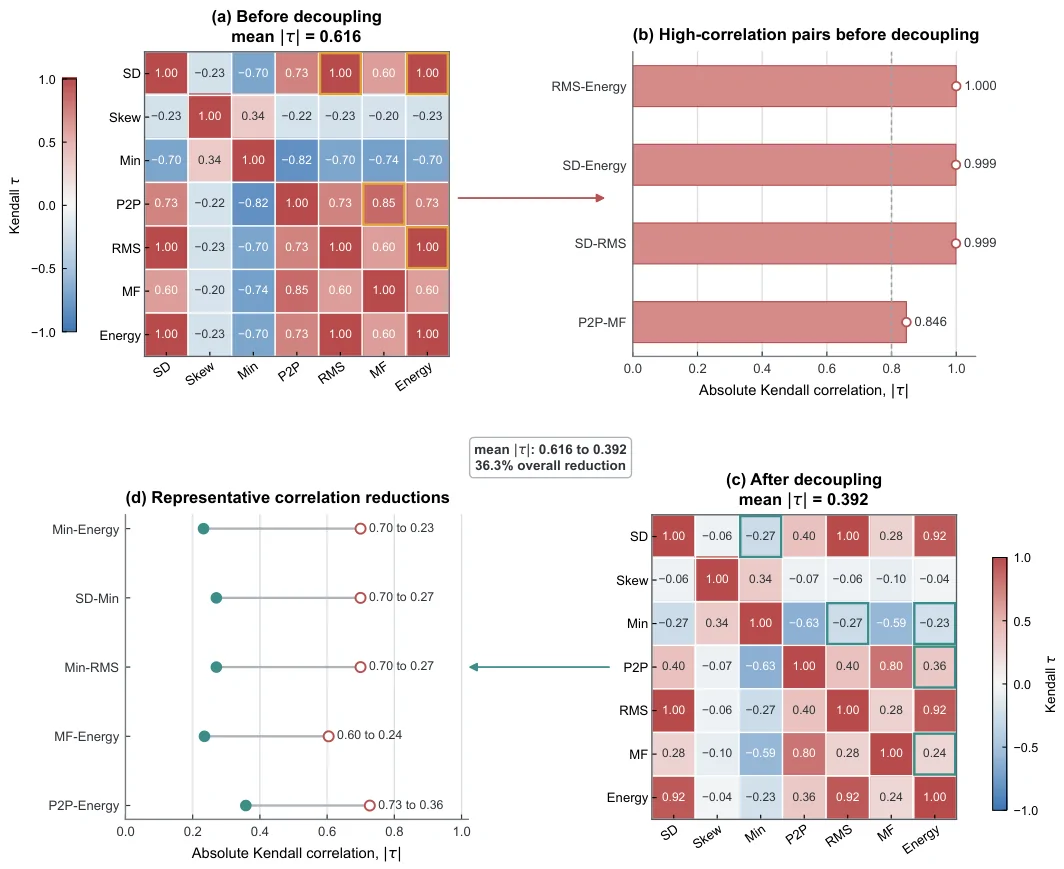
Comparison of Kendall correlation matrices before and after attribute decoupling.

The selected input attributes are first standardized using statistics calculated exclusively from the training set and then mapped into the decoupled feature space through the correlation-aware attribute transformation described in Section 3.1. The resulting transformation matrix is fixed and directly applied to the test samples to prevent information leakage. As shown in Figure 4a,b, several attributes exhibit strong redundancy before decoupling, with the correlations among SD, RMS, and Energy approaching 1.0 and that between P2P and MF reaching 0.846. After transformation, the mean absolute off-diagonal Kendall correlation decreases from 0.616 to 0.392, corresponding to an overall reduction of 36.3%, as illustrated in Figure 4c. Representative examples in Figure 4d further show that the absolute correlations of Min–Energy, SD–Min, Min–RMS, MF–Energy, and P2P–Energy are substantially reduced. These results indicate that the transformation suppresses, rather than completely eliminates, redundant dependence among the attributes. Consequently, correlated information is less likely to be repeatedly involved in rule activation and evidential reasoning, thereby limiting the propagation of attribute disturbances while retaining the diagnostic information required by the subsequent CD-IBRB model.

To avoid information leakage and uncontrolled condition-dependent transformation, the correlation-aware decoupling matrix is estimated exclusively from the source training data and is then fixed when transforming the test data. Thus, no target-domain samples or target labels are involved in the construction of the transformation matrix. The decoupling procedure is applied to standardized time-domain diagnostic features rather than to raw vibration signals or physical mechanism variables. Moreover, the optimization objective constrains the decoupling matrix to remain close to the identity matrix, thereby limiting unnecessary deviations from the original feature representation. Kendall rank correlation is adopted to reduce redundant monotonic dependence among attributes without imposing a linear correlation assumption. Therefore, the transformed attributes are used as less-redundant diagnostic-evidence coordinates for rule reasoning, rather than as direct physical replacements for the original features.

Step 3: Construction of Referential Points, Intervals, and the Initial Belief Table

After attribute decoupling, referential points are generated independently for each transformed attribute according to its distribution characteristics. For approximately normal attributes, the referential points are distributed around the mean, whereas empirical quantiles are used for skewed or irregular attributes. Adjacent referential points are then combined to form referential intervals. This attribute-specific construction strategy enables different attributes to adopt appropriate interval granularities and avoids the rule explosion caused by the Cartesian product of multiple reference values. The initial parameter settings of the CD-IBRB model are summarized in Table 2.

Based on these referential intervals, the initial belief table is constructed using the class distribution of the training samples. For each interval rule, the initial belief degree assigned to a fault class is determined by the proportion of training samples belonging to that class within the corresponding interval. A smoothing coefficient is introduced to avoid zero belief degrees, after which the belief distribution is normalized. The resulting belief table establishes an interpretable relationship between each attribute interval and the four bearing health conditions. The initial parameters of the CD-IBRB model are presented in Table 2 and subsequently optimized using the P-CMA-ES algorithm.

Step 4: Parameter Optimization of the CD-IBRB Model

(1) P-CMA-ES Parameter Settings

P-CMA-ES is employed to jointly optimize the consequent belief degrees, rule reliabilities, and rule weights by minimizing the cross-entropy loss on the training set. The initial belief degrees are determined from the class distributions of the training samples within the corresponding referential intervals, as defined in Equation (35). The rule reliabilities and rule weights are initially set to 1. These parameters are concatenated to form the initial mean vector of P-CMA-ES, while the initial covariance matrix is set to the identity matrix. The main optimizer settings are summarized in Table 3.

The optimization is terminated when the predefined maximum number of generations is reached. Although the cross-entropy loss generally becomes stable during the evolutionary process, premature termination may prevent the optimizer from sufficiently exploring the feasible parameter space. Therefore, the maximum number of generations is set to 200 to balance optimization performance and computational cost. Throughout the optimization process, the best feasible solution is retained according to the minimum cross-entropy loss. The covariance matrix update coefficients are calculated using the standard P-CMA-ES parameterization based on the 420-dimensional parameter vector and the effective number of selected offspring.

(2) Optimized CD-IBRB Parameters

**Figure 5:**
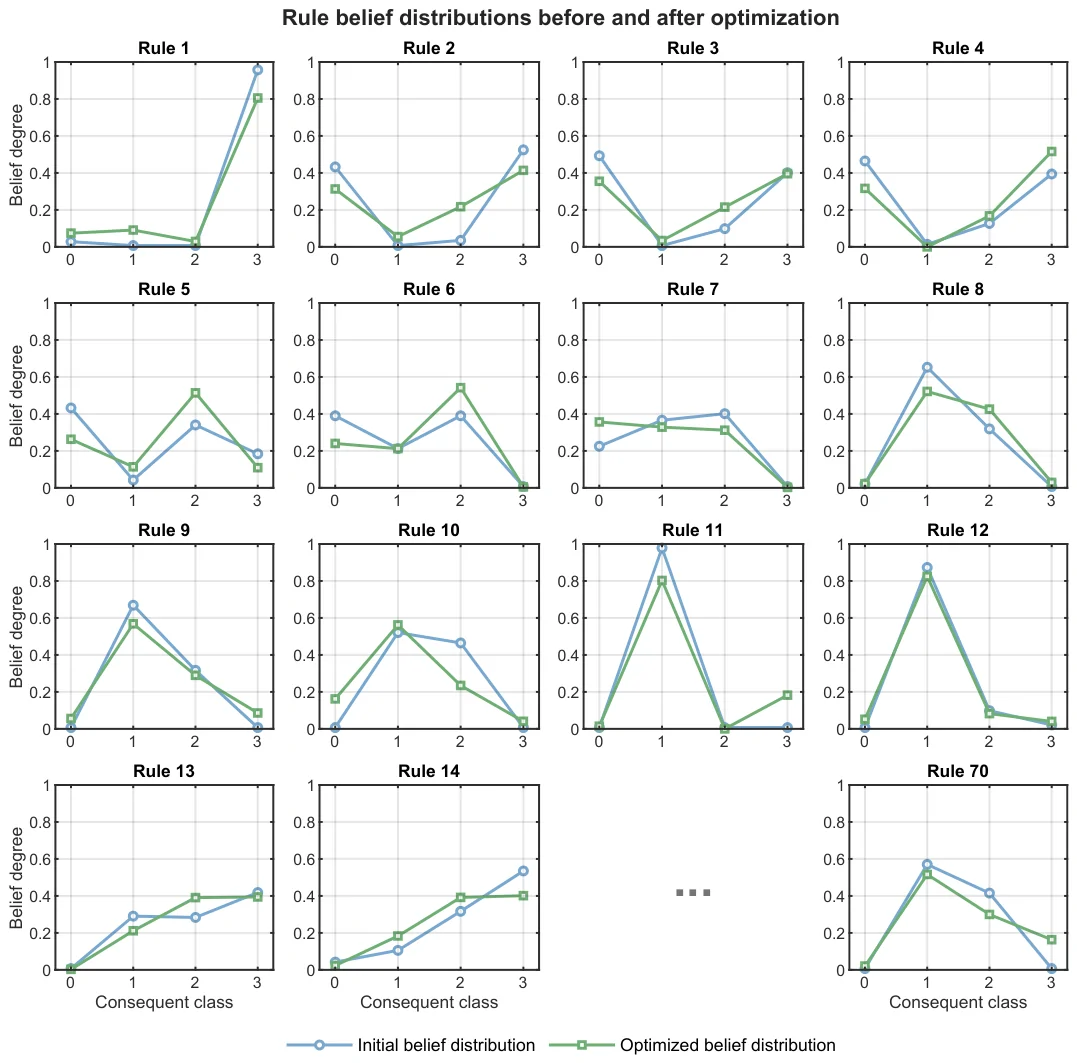
Rule belief distributions of the CD-IBRB model before and after optimization.

Based on the above configuration, P-CMA-ES minimizes the cross-entropy loss on the training set to jointly optimize the consequent belief degrees, rule reliabilities, and rule weights. During optimization, the normalization and boundary constraints are enforced to ensure parameter validity and preserve the explicit interval-rule structure. The best feasible parameter vector obtained during the optimization process is then used to construct the final CD-IBRB model. The optimized model parameters are reported in Table 4, while the changes in the rule belief distributions before and after optimization are illustrated in Figure 5.

### 5.3. Experimental Analysis of the CD-IBRB

This section evaluates the fault diagnosis performance of the CD-IBRB model using a bearing dataset collected under practical operating conditions. The model is assessed from both overall and class-specific perspectives using accuracy, macro-averaged F1 score, and the precision and recall of each fault class. These complementary metrics provide a comprehensive evaluation of the diagnostic effectiveness and class-balanced performance of CD-IBRB under cross-condition scenarios.

(1) Parameter Optimization Analysis of the CD-IBRB Model

**Figure 6:**
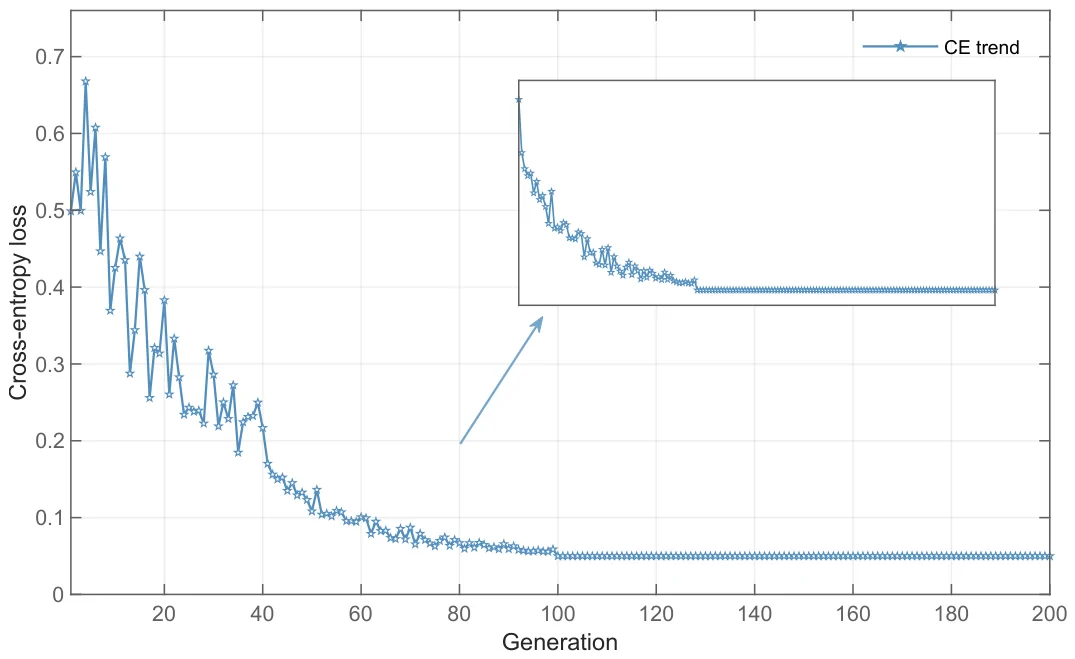
Convergence curve of the cross-entropy loss during CD-IBRB optimization.

As shown in Figure 6, the cross-entropy loss decreased progressively during parameter optimization, although noticeable fluctuations occurred in the early generations. After approximately 100 generations, the curve gradually stabilized, indicating that P-CMA-ES had approached a stable optimum under the current parameter settings and that further iterations produced only limited improvement. Figure 5 compares the rule belief distributions before and after optimization. The optimized distributions exhibited clearer differentiation among the consequent classes, demonstrating that the joint adjustment of belief degrees, rule reliabilities, and rule weights strengthened the diagnostic evidence represented by the activated rules. These results illustrate the effectiveness of parameter optimization in improving the convergence behavior and output discriminability of the CD-IBRB model.

(2) Analysis of Bearing Fault Diagnosis Results Using CD-IBRB

The bearing dataset used in this experiment followed the cross-condition configuration described in Section 5.1. Detailed information on the operating conditions, fault categories, and sample distribution is summarized in Table 1 of Section 5.1.

**Figure 7:**
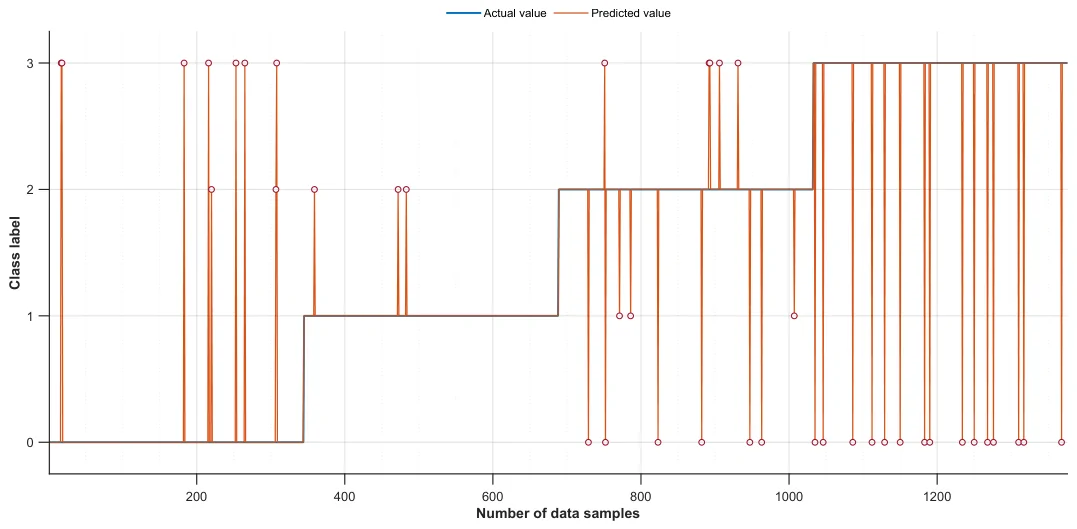
Bearing fault diagnosis results of the CD-IBRB model.

As shown in Figure 7 and Table 5, the CD-IBRB model achieved an accuracy of 0.9702 and a macro-averaged F1 score of 0.9702, indicating both high overall diagnostic accuracy and balanced classification performance across the four bearing conditions. In particular, ORF achieved the highest precision and recall, both reaching 0.9912, while the precision and recall for IRF were 0.9850 and 0.9593, respectively. For BF, these values reached 0.9648 and 0.9563. Although the precision for NC was relatively lower at 0.9410, its recall remained high at 0.9738, demonstrating that most normal samples were correctly identified. The close agreement between accuracy and macro-averaged F1 score further indicates that the diagnostic performance was not dominated by a particular class. These results suggest that attribute decoupling reduces the repeated activation of correlated evidence, while interval-rule reasoning and parameter optimization improve the discrimination of belief distributions among different fault classes. Consequently, CD-IBRB provides accurate and relatively balanced recognition of different bearing health conditions.

### 5.4. Ablation Experiments

**Figure 8:**
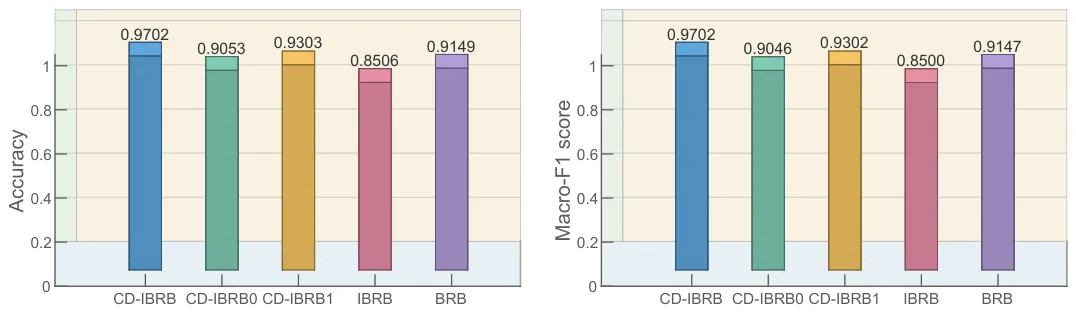
Ablation analysis of the CD-IBRB model.

To quantify the contributions of attribute decoupling and parameter optimization, ablation experiments were conducted on the same bearing dataset using an identical training and test protocol. The complete CD-IBRB model incorporates both attribute decoupling and P-CMA-ES optimization. Four variants were considered for comparison. CD-IBRB0 retained the attribute decoupling procedure but used the initial rule parameters without optimization. CD-IBRB1 removed attribute decoupling while retaining P-CMA-ES optimization. IBRB excluded both attribute decoupling and parameter optimization. In addition, an optimized conventional BRB model was introduced to compare the interval-rule structure with the traditional Cartesian-product rule construction. To control the number of combinatorial rules, the conventional BRB used only the two highest-ranked attributes identified using the feature-selection method described in Section 4.1. All models were evaluated using the same data partition, preprocessing procedure, and evaluation metrics. The corresponding ablation results are presented in Figure 8 and Table 6.

Contribution Analysis of CD-IBRB Components:

As shown in Figure 8 and Table 6, CD-IBRB achieved the highest accuracy and macro-averaged F1 score, both reaching 0.9702. Removing parameter optimization reduced the accuracy to 0.9053, whereas removing attribute decoupling decreased it to 0.9303, indicating that both components contributed to diagnostic performance, with parameter optimization providing the larger individual improvement. When both components were removed, IBRB achieved only 0.8506 accuracy and 0.8500 macro-F1. These results indicate that attribute decoupling reduces redundant evidence among correlated inputs, while parameter optimization improves the discriminability of interval belief rules. Their combination therefore enables more accurate and class-balanced fault diagnosis.

The optimized conventional BRB achieved an accuracy of 0.9149 and a macro-averaged F1 score of 0.9147, which were lower than those of CD-IBRB by 5.53 and 5.55 percentage points, respectively. In the conventional BRB, antecedent rules are constructed through the Cartesian product of attribute referential values. Consequently, incorporating more attributes causes the number of rules and parameters to increase exponentially, substantially increasing the computational burden and weakening model interpretability. Restricting the model to only a few attributes can control rule-base complexity but may discard complementary physical information related to signal dispersion, impulsiveness, amplitude variation, and energy. By constructing interval rules independently for each attribute, CD-IBRB can incorporate all seven selected attributes while allowing the rule-base size to grow additively rather than exponentially. Therefore, the proposed model achieves a more favorable balance between diagnostic information coverage, rule-base complexity, and diagnostic performance.

### 5.5. Comparative Experiments

To further evaluate the effectiveness and comparative performance of CD-IBRB, seven representative baseline models were selected. These baselines included three BRB variants, namely the approximate belief rule base (ABRB), extended belief rule base (EBRB), and hierarchical belief rule base (HBRB), as well as four classical data-driven models: decision tree (DT), back-propagation neural network (BPNN), logistic regression (LR), and naive Bayes (NB). The selected methods cover different modeling paradigms, including uncertainty reasoning, extended and hierarchical rule organization, tree-based classification, neural learning, linear discriminative modeling, and probabilistic inference. All models were evaluated using the same cross-condition dataset partition, feature-processing procedure, and evaluation metrics. Accuracy, macro-averaged F1 score, and class-wise precision and recall were adopted to assess their overall and class-balanced diagnostic performance.

**Figure 9:**
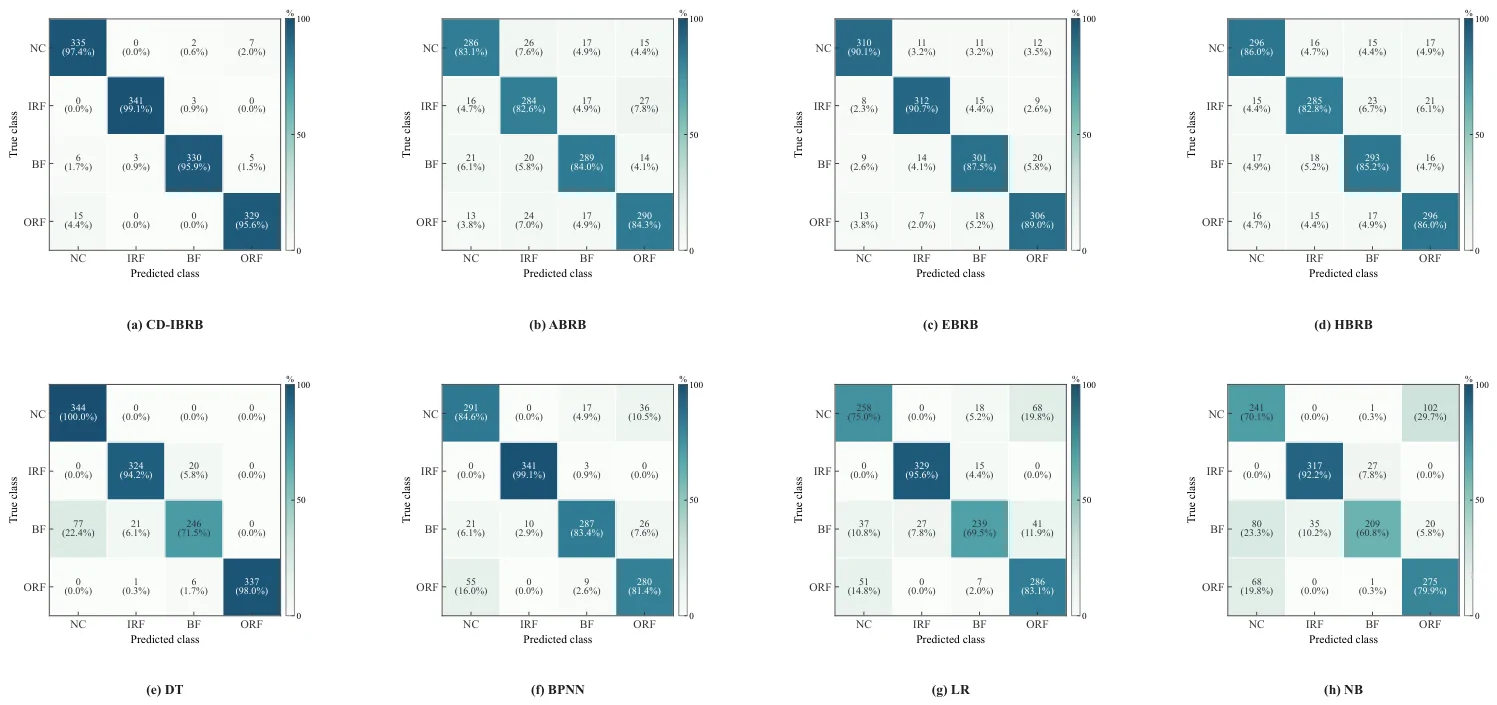
Comparison of bearing fault classification results across different models.

As shown in Figure 9 and Table 7, CD-IBRB achieved the best overall performance among all the evaluated models, with an accuracy of 0.9702 and a macro-averaged F1 score of 0.9703. Only 41 of the 1376 test samples were misclassified. DT produced substantial errors for IRF, whereas EBRB, ABRB, and HBRB showed more dispersed deviations across the four categories. BPNN, LR, and NB exhibited stronger class-dependent biases, indicating weaker performance consistency.

At the class level, ORF achieved the highest precision and recall, both reaching 0.9912, which may be attributed to its relatively stable and distinguishable impact characteristics. The NC precision was comparatively lower at 0.9410, while its recall remained high at 0.9738. This indicates that most NC samples were correctly recognized, although several weak fault samples with normal-like vibration characteristics were assigned to NC under changing operating conditions. IRF achieved a precision of 0.9850 and a recall of 0.9593, while BF obtained a precision of 0.9648 and a recall of 0.9563. The slightly lower recall values for IRF and BF may result from load-induced variations in fault impulses and the greater overlap of their time-domain features with other conditions. Nevertheless, all class-wise precision and recall values exceeded 0.94, demonstrating balanced diagnostic performance. These results are consistent with the CD-IBRB modeling mechanism. Attribute decoupling reduces the repeated use of correlated evidence, while attribute-wise interval rules accommodate feature fluctuations without Cartesian-product rule expansion. Evidential reasoning integrates uncertain rule evidence, and P-CMA-ES further optimizes the belief degrees, rule reliabilities, and rule weights. Their combination enables CD-IBRB to distinguish the four bearing conditions more accurately and consistently under cross-condition scenarios.

(1) Comparison with BRB Variant Models

Among the BRB-based models, CD-IBRB achieved the best diagnostic performance, with an accuracy of 0.9702 and a macro-averaged F1 score of 0.9703. Compared with ABRB, EBRB, and HBRB, its accuracy increased by 16.2%, 8.6%, and 14.1%, respectively, with similar relative improvements in macro-averaged F1 score. At the class level, the precision of CD-IBRB ranged from 0.9410 to 0.9912, while its recall ranged from 0.9563 to 0.9912. These consistently high class-wise metrics indicate that its overall advantage was not dominated by a single fault category.

Among the three BRB variants, EBRB achieved the strongest performance, with both accuracy and macro-averaged F1 score reaching 0.8932. Although ABRB, EBRB, and HBRB improve conventional BRB modeling through approximate reasoning, extended rule representation, or hierarchical organization, their formulations do not explicitly reduce redundant correlations among vibration attributes. Consequently, correlated diagnostic evidence may be repeatedly involved in rule activation and evidential aggregation. In addition, approximate or hierarchical rule structures may reduce model complexity but could weaken the representation of fine-grained relationships between physical attributes and fault states.

In contrast, CD-IBRB first transforms the selected attributes into a less-correlated feature space and subsequently constructs interval rules independently for each attribute. This structure represents all seven diagnostic attributes using 70 interval rules without introducing Cartesian-product rule expansion. The complementary evidence provided by the activated interval rules is then aggregated through evidential reasoning, while P-CMA-ES jointly optimizes the belief degrees, rule reliabilities, and rule weights. The performance improvements over the three BRB variants support the effectiveness of integrating attribute decoupling, interval-rule construction, and parameter optimization for cross-condition bearing fault diagnosis.

(2) Comparison with Machine Learning Models

CD-IBRB also outperformed all four data-driven baseline models in terms of accuracy and macro-averaged F1 score. DT was the strongest data-driven competitor, achieving an accuracy of 0.9092 and a macro-averaged F1 score of 0.9067. In comparison, CD-IBRB improved these two metrics by approximately 6.7% and 7.0%, respectively. BPNN achieved an accuracy of 0.8714, followed by LR and NB with accuracies of 0.8081 and 0.7573, respectively. Their macro-averaged F1 scores exhibited a similar ranking, confirming that the advantage of CD-IBRB was maintained across the four bearing conditions.

DT captured the principal classification boundaries but showed relatively weak recognition of IRF, for which its recall decreased to 0.7151. This result may be associated with the hard feature-partitioning mechanism of decision trees, which can be sensitive to changes in feature distributions across operating conditions. BPNN achieved high precision and recall for ORF, reaching 0.9715 and 0.9912, respectively, but exhibited weaker performance for Normal, IRF, and BF. This imbalance indicates that its learned nonlinear mapping did not generalize equally across all classes. LR was constrained by its linear decision boundaries, particularly for overlapping fault distributions, resulting in an IRF recall of 0.6947 and a BF precision of 0.7240. NB exhibited the lowest overall performance, with an IRF recall of only 0.6075 and a Normal precision of 0.6195. These results indicate that its conditional-independence and class-distribution assumptions were not fully consistent with the correlated and nonlinear characteristics of bearing vibration features. By combining correlation-aware attribute transformation, interval-based uncertainty representation, and evidential reasoning, CD-IBRB maintained more balanced diagnostic performance across the four bearing conditions.

Unlike these data-driven models, CD-IBRB represents diagnostic knowledge through explicit interval rules, with each rule linking a physically meaningful attribute interval to a belief distribution over the four bearing conditions. This structure allows engineers to inspect the activated rules, rule reliabilities, rule weights, and output belief degrees associated with each diagnostic decision. Moreover, attribute decoupling reduces redundant feature information, evidential reasoning integrates complementary diagnostic evidence, and P-CMA-ES improves the discrimination of the output belief distributions. Therefore, the comparative results demonstrate that CD-IBRB provides a more favorable combination of diagnostic accuracy, class-balanced performance, uncertainty representation, and model interpretability than the selected data-driven baselines.

### 5.6. Cross-Load Generalization Analysis

To evaluate the cross-load generalization capability of the proposed model, a series of experiments was conducted using bearing samples with a fault width of 0.2 mm and a rotational speed of 2000 r/min. Four load conditions, namely 0, 20, 40, and 60 N, were considered. The samples collected under 40 N were used exclusively for model training, whereas those collected under 0, 20, and 60 N were independently employed as target-domain test sets. This configuration covers both moderate load variations and the challenging transition from 40 N to the unloaded condition of 0 N, thereby providing a rigorous evaluation of model adaptability to load-induced distribution shifts.

**Figure 10:**
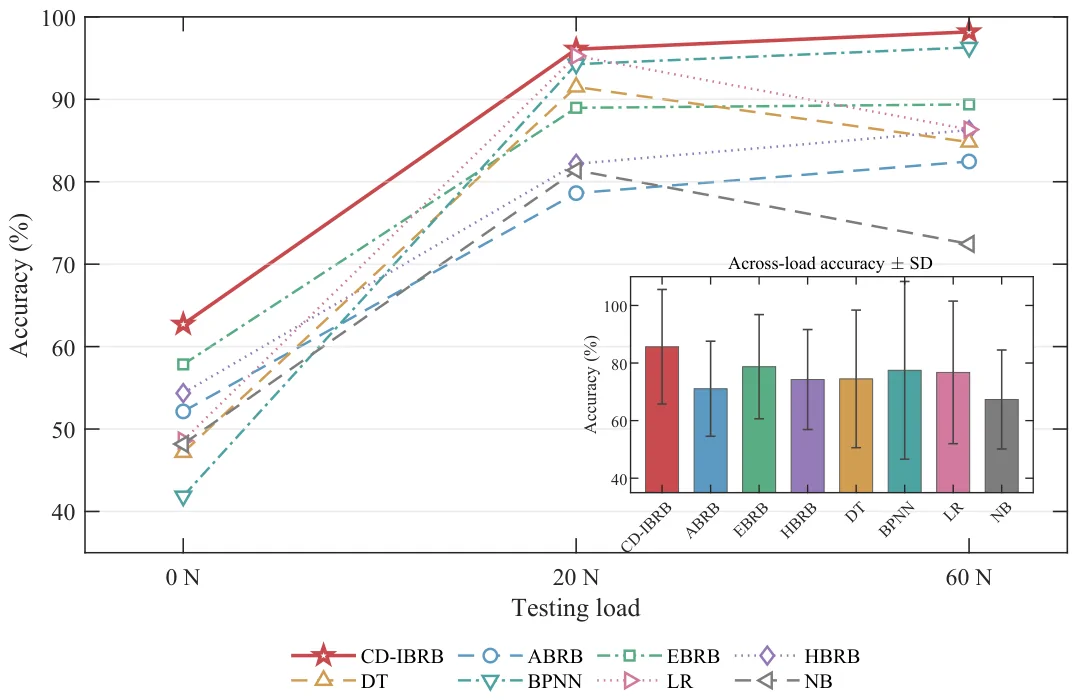
Cross-load diagnostic performance of different models.

To prevent data leakage and direct overlap between adjacent samples, only vibration signals acquired from the first measurement channel were used. The continuous signals under each load condition were segmented using a sliding window with a length of 2048 sampling points and a step size of 3048 sampling points. Since the step size exceeded the window length, no sampling points were shared between adjacent segments, thereby reducing dependence caused by overlapping signal windows. All comparative models followed the same training and testing protocol, and target-domain labels were used only for final performance evaluation. The diagnostic accuracies of the different models under each target load are presented in Figure 10. Their overall performance, expressed as the mean ± cross-load standard deviation (SD) across the three transfer tasks, is summarized in Table 8.

As shown in Figure 10 and Table 8, all models exhibit substantial performance degradation when transferred from the 40 N source condition to the 0 N target condition, indicating that the abrupt load reduction causes a pronounced shift in the distributions of vibration features. Nevertheless, CD-IBRB achieves the highest accuracies under the 0, 20, and 60 N conditions, reaching 62.72%, 96.08%, and 98.18%, respectively. This advantage can be attributed to correlation decoupling, which reduces the repeated propagation of disturbances among correlated attributes, and interval-rule reasoning, which provides tolerance to feature fluctuations near class boundaries. Parameter optimization further improves the consistency between the belief-rule representation and the observed fault patterns.

In comparison, DT is sensitive to changes in feature-splitting boundaries, while LR is limited by its linear decision structure. NB relies on the conditional-independence assumption, which is difficult to satisfy for correlated vibration features. Although BPNN provides strong performance under the 20 and 60 N conditions, its learned feature-to-class mapping remains sensitive to the severe distribution discrepancy under 0 N. The BRB variants also exhibit lower cross-load performance because they do not simultaneously account for attribute correlation and interval uncertainty. Although CD-IBRB cannot completely eliminate the effect of an extreme domain shift, the overall results demonstrate its stronger cross-load adaptability and more balanced diagnostic performance.

It should be noted that Accuracy ± SD and Macro-Recall ± SD are identical in Table 8 because each target dataset contains the same number of samples for every fault class. Under this class-balanced setting, overall accuracy is mathematically equivalent to the unweighted average of class-wise recall. Consequently, the two metrics have identical values in each transfer task and therefore produce the same cross-load mean and SD.

### 5.7. Disturbance Experiments

**Figure 11:**
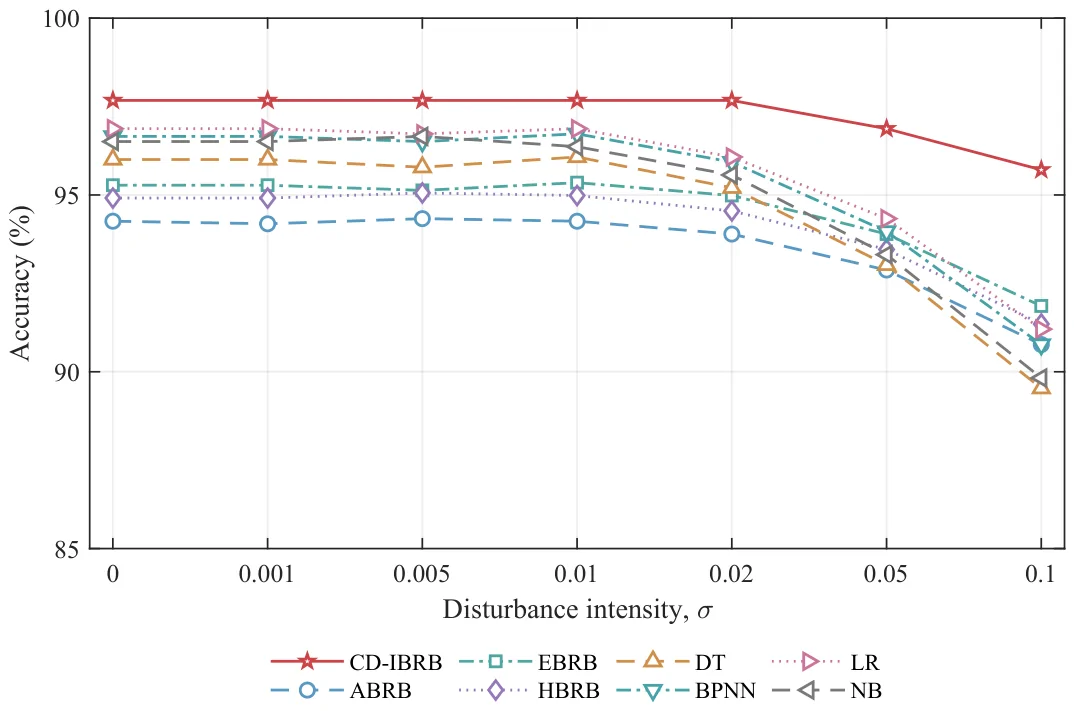
Diagnostic accuracy of different models under varying disturbance intensities.

To further evaluate the robustness of the proposed method under complex noisy environments, a disturbance injection experiment was conducted. Bearing vibration data collected at a rotational speed of 3000 r/min with a fault size of 0.2 mm were employed. The data obtained under the 20 N load condition were used for model training, whereas those collected under the 40 N load condition were used for testing, thereby introducing a cross-load distribution shift. To simulate measurement noise and random interference commonly encountered in practical industrial environments, white noise was injected into the input attributes of the training data. In addition to the noise-free condition (σ=0), six disturbance levels were considered, namely σ∈{0.001,0.005,0.01,0.02,0.05,0.1}. This experimental design enables a systematic assessment of the diagnostic stability and noise resistance of each model as the disturbance intensity increases. The diagnostic accuracies of the compared models under different disturbance levels are illustrated in Figure 11, and the corresponding quantitative results are summarized in Table 9.

As shown in Figure 11 and Table 9, the diagnostic performance of most models remains relatively stable under weak disturbances but gradually deteriorates as the disturbance intensity increases. When σ≤0.02, CD-IBRB maintains an accuracy of 97.67%, indicating that weak noise has little influence on its diagnostic results. Some baseline models exhibit slight accuracy increases at individual disturbance levels. For example, EBRB increases from 95.28% at σ=0.001 to 95.35% at σ=0.01, while BPNN increases from 96.66% to 96.73%. These small non-monotonic fluctuations suggest that weak random disturbances may move a limited number of boundary samples toward the correct decision regions. Nevertheless, these local improvements are minor and do not represent an overall increase in robustness.

When the disturbance intensity increases to σ=0.05 and σ=0.1, the performance differences become more pronounced. At σ=0.1, the accuracies of DT, BPNN, LR, and NB decrease to 89.53%, 90.77%, 91.21%, and 89.93%, respectively, whereas CD-IBRB retains an accuracy of 95.71%. EBRB achieves the highest baseline accuracy of 91.86%, which remains 3.85 percentage points lower than that of CD-IBRB. This indicates that conventional data-driven models are more sensitive to noise-induced feature shifts and decision-boundary changes. By reducing the repeated propagation of disturbances among correlated attributes and improving tolerance to feature fluctuations through interval rules and evidential reasoning, CD-IBRB exhibits a slower performance decline and greater diagnostic stability. Moreover, the BRB variants generally show milder performance fluctuations than the data-driven models, further demonstrating the inherent robustness of belief-rule-based modeling in handling uncertain and disturbed information.

### 5.8. Interpretability and Computational Complexity Analysis of the CD-IBRB Model

(1) Interpretability Analysis

To illustrate the rule-level traceability of CD-IBRB, a correctly classified inner-ring fault sample was selected from the 40 N test condition. For this sample, seven interval rules were activated, corresponding to the seven decoupled input attributes. Among them, Rule 56 produced the largest effective contribution to the ER aggregation. The corresponding reasoning information is summarized in Table 10.

The seventh decoupled attribute is mainly determined by the shape factor, while retaining smaller contributions from the other selected attributes. Its transformation can be expressed approximately as $X_{7}^{*}=-0.0748z_{Energy}-0.0892z_{SD}-0.1079z_{MF}-0.0892z_{RMS}-0.1126z_{IF}-0.1124z_{P2P}+0.9050z_{SF}.$

As shown in Table 10, the optimized consequent belief distribution of Rule 56 assigns the highest belief degree to IRF (0.7950). This distribution represents the diagnostic evidence provided by Rule 56 rather than the final model output. After Rule 56 and the other six activated attribute-wise rules are aggregated using the ER algorithm, the final output belief distribution for the selected sample is obtained as {NC: 0.0004, IRF: 0.9992, BF: 0.0002, ORF: 0.0001}. Therefore, the sample is diagnosed as an inner-ring fault.

This example demonstrates that the diagnostic result can be traced through the decoupled attribute value, activated interval, rule reliability, rule weight, rule consequent, and final aggregated belief distribution. Nevertheless, because each decoupled attribute is a linear combination of the original features, the proposed method provides rule-level and decision-level traceability rather than a complete physical interpretation of every transformed attribute. This distinction avoids overstating the interpretability of the model.

(2) Computational Complexity Analysis

The computational complexity of CD-IBRB is summarized in Table 11.

Here, *n*, *M*, *K*, and *C* denote the numbers of training samples, input attributes, referential points, and output classes, respectively. Moreover, *G* and λ represent the number of P-CMA-ES generations and offspring population size, while D=L(C+2) is the number of optimized parameters and L=M(K−1) is the number of rules.

In the present experiment, M=7, K=11, C=4, and L=70, resulting in 420 optimized parameters, including 280 belief degrees, 70 rule reliabilities, and 70 rule weights. Although P-CMA-ES introduces a relatively high offline optimization cost, this procedure is performed only during model training. During inference, each attribute activates only one interval rule; therefore, only seven of the 70 rules participate in ER aggregation for each sample. This attribute-wise structure avoids the exponential rule growth of a combinatorial BRB and provides efficient online reasoning while preserving a transparent and traceable rule structure.

Compared with conventional combinatorial BRB models, CD-IBRB reduces the number of rules from exponential growth to a linear form, L=M(K−1), substantially alleviating rule explosion as the number of attributes increases. Although DT, LR, and NB may incur lower computational costs, they cannot simultaneously provide explicit interval rules, belief distributions, and traceable evidential reasoning. Compared with BPNN, CD-IBRB requires additional offline parameter optimization but offers a more transparent diagnostic process. Therefore, CD-IBRB achieves a practical balance among computational efficiency, uncertainty representation, and model interpretability.

### 5.9. Generalization Experiments

To further examine the generalization capability of CD-IBRB beyond cross-condition bearing fault diagnosis, five publicly available multiclass benchmark datasets, namely Abalone, Ecoli, Iris, New-Thyroid, and Shuttle, were selected from the UCI Machine Learning Repository and the KEEL Dataset Repository. These datasets represent diverse application domains, including marine organism age classification, protein localization, plant species recognition, thyroid disease diagnosis, and aerospace system monitoring. They also exhibit substantial differences in sample size, attribute dimensionality, number of classes, class distribution, and attribute correlation. Such heterogeneity provides a suitable basis for evaluating the adaptability of CD-IBRB under different data structures and degrees of class imbalance.

For each dataset, stratified sampling was used to allocate 70% of the samples to the training set and the remaining 30% to the test set. To avoid data leakage, all data-dependent operations, including preprocessing, attribute decoupling, referential point and interval construction, belief table initialization, and parameter optimization, were conducted exclusively using the training data. The learned transformation parameters and optimized model were then fixed and applied to the corresponding test set. All comparative models used identical data partitions and evaluation metrics to ensure a consistent assessment. The main characteristics and sample distributions of the five datasets are summarized in Appendix A.

**Figure 12:**
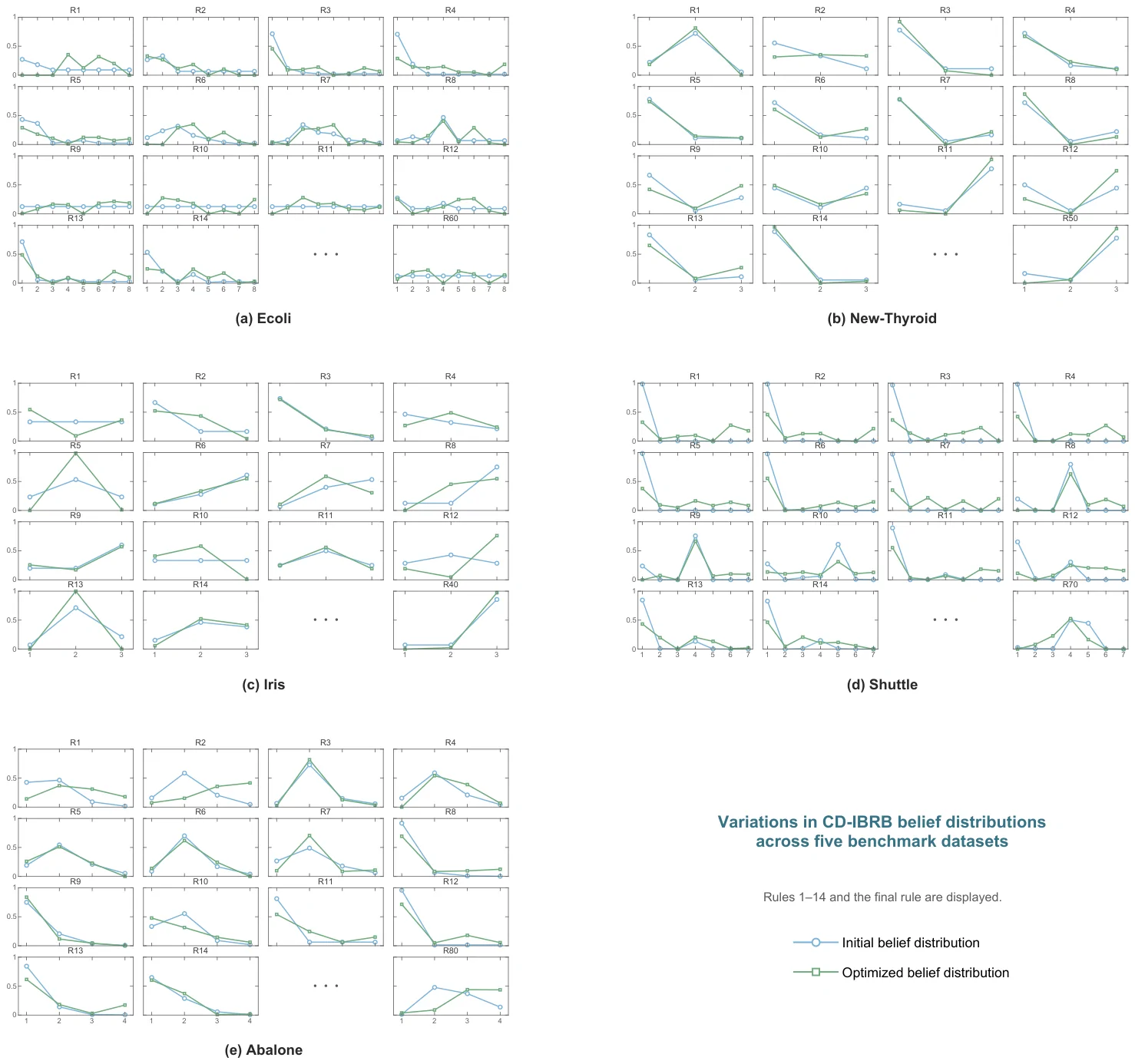
Comparison of initial and optimized belief distributions across different benchmark datasets.

Given the differences in valid attribute dimensionality and data structure, a dataset-specific rule base was constructed for each benchmark dataset. The resulting CD-IBRB models contained 80, 60, 40, 50, and 70 rules for Abalone, Ecoli, Iris, New-Thyroid, and Shuttle, respectively. These differences were primarily determined by the number of retained input attributes and the corresponding interval construction rather than by sample size alone. As shown in Figure 12, parameter optimization modified the initial belief distributions according to the class characteristics of each dataset. The resulting changes demonstrate the data-driven calibration of CD-IBRB while preserving its explicit rule-based representation.

**Figure 13:**
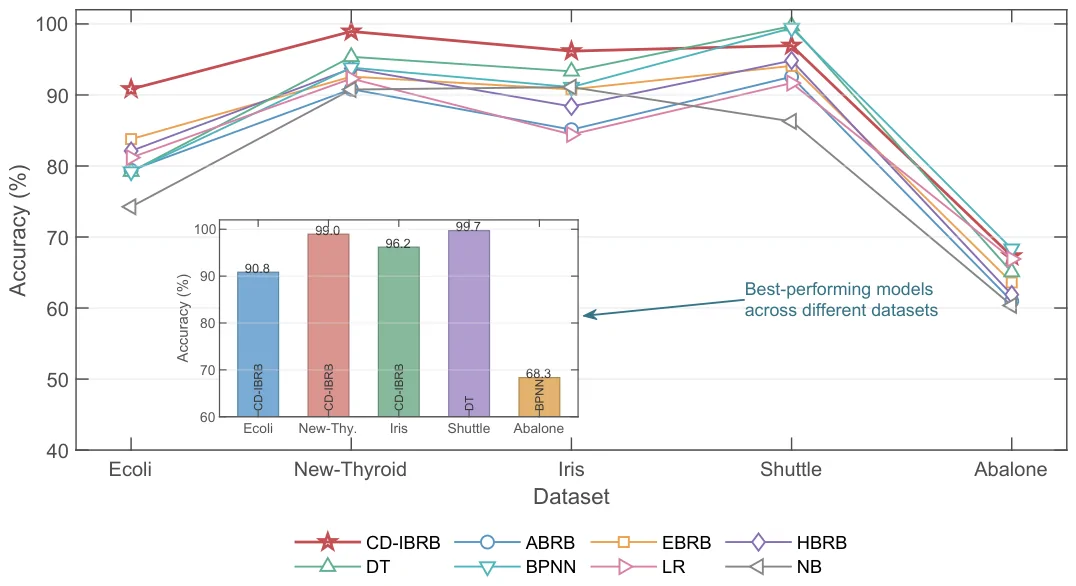
Performance comparison of different models across benchmark datasets.

As shown in Figure 13 and Appendix B, CD-IBRB exhibited the strongest overall generalization performance across the five benchmark datasets. It achieved the highest accuracy on Ecoli (90.83%), New-Thyroid (98.95%), and Iris (96.18%), while consistently outperforming the three BRB variants, namely ABRB, EBRB, and HBRB, on all datasets. Its average accuracy reached 90.04%, exceeding those of DT (86.54%), BPNN (86.39%), and the best-performing BRB variant, EBRB (84.97%). Although DT and BPNN achieved higher accuracy on Shuttle, and BPNN slightly outperformed CD-IBRB on Abalone, CD-IBRB remained competitive in both cases. The macro-averaged metrics in Appendix B further demonstrated relatively balanced classification performance on Ecoli, New-Thyroid, Iris, and Shuttle. However, the lower macro-F1 score and macro recall obtained on Abalone indicate that severe class imbalance and complex class boundaries remain challenging. Overall, these results support the adaptability of CD-IBRB to datasets with different sample sizes, feature structures, and class distributions, while its consistent advantage over the BRB variants is consistent with the intended benefits of attribute decoupling and interval-based rule construction.

## 6. Discussion

The results indicate that CD-IBRB provides an effective and traceable framework for cross-condition bearing fault diagnosis. It achieved an accuracy of 0.9702 and a macro-averaged F1 score of 0.9703 in the primary experiment. The mean absolute Kendall correlation decreased from 0.616 to 0.392 after attribute decoupling. This reduction limits the repeated use of correlated vibration information during rule activation and evidential aggregation. The ablation results further support the complementary contributions of decoupling and parameter optimization. Removing these components reduced the accuracy to 0.9303 and 0.9053, respectively. The differences among fault categories may reflect their vibration characteristics. ORF produced relatively stable impact patterns and achieved the highest precision and recall. In contrast, some weak fault samples retained normal-like characteristics, resulting in a lower NC precision of 0.9410.

Recent international studies based on deep learning, self-supervised learning, physics-informed modeling, and domain adaptation have improved feature learning and cross-condition diagnosis. These methods are effective at extracting complex nonlinear representations but generally provide limited rule-level traceability. CD-IBRB instead represents diagnostic evidence through explicit interval rules and belief distributions. Compared with ABRB, EBRB, and HBRB, it also reduces attribute correlation before rule construction. Its attribute-wise structure generates 70 rules additively and activates only seven rules for each sample, avoiding the Cartesian-product growth of conventional BRBs. However, the model still contains 420 optimized parameters and requires relatively costly offline P-CMA-ES optimization. Its main advantage therefore lies in balancing diagnostic performance, uncertainty representation, rule-base complexity, and reasoning traceability rather than minimizing training cost.

The supplementary experiments further define the applicability of CD-IBRB. In the cross-load experiment, it achieved accuracies of 62.72%, 96.08%, and 98.18% under the 0, 20, and 60 N target conditions, respectively. The lower performance for the 40 N-to-0 N transfer indicates that severe domain shifts remain challenging. In the disturbance experiment, CD-IBRB retained an accuracy of 95.71% at a noise intensity of 0.1, exceeding the strongest baseline by 3.85 percentage points. Across five benchmark datasets, it achieved a mean accuracy of 0.9004 and consistently outperformed the compared BRB variants. Nevertheless, DT and BPNN performed better on Shuttle, while BPNN slightly outperformed CD-IBRB on Abalone. These findings indicate broad adaptability but do not support universal superiority over all data-driven models.

The current dataset contains four discrete bearing conditions representing health states and fault locations rather than consecutive stages of damage accumulation. It does not include continuously labelled observations covering the progression from initial wear to fully developed damage or explicitly defined pre-boundary and boundary states. Therefore, the present results validate CD-IBRB for fault-category classification but do not establish its effectiveness across all stages of bearing degradation or for incipient-fault and boundary-state diagnosis. Although a dispersed output belief distribution may indicate increased diagnostic uncertainty, it cannot by itself be interpreted as a specific physical damage stage, pre-boundary state, or early-fault condition without corresponding degradation labels and inspection evidence. Future work will employ run-to-failure datasets with explicitly labelled degradation stages to evaluate the identification of initial, pre-boundary, boundary, developing, and fully developed bearing damage.

Several limitations remain. The bearing experiments mainly used data from one laboratory platform, one sensor channel, and restricted operating conditions. White-noise injection also cannot fully reproduce startup, shutdown, abrupt load changes, compound faults, or sensor degradation. Moreover, the decoupled attributes are less physically intuitive than the original features, and performance may depend on the selected decoupling strength and interval configuration. Future research will therefore evaluate CD-IBRB using multisensor field data and continuously varying operating conditions. Structure-preserving decoupling, adaptive interval construction, uncertainty calibration, dynamic rule updating, and online optimization will also be investigated to improve physical interpretability, computational efficiency, and industrial applicability.

## 7. Conclusions

This study proposed a correlation-decoupled interval belief rule base for cross-condition bearing fault diagnosis. The primary objective was to reduce the repeated use of correlated vibration evidence, avoid the combinatorial rule growth of conventional BRB models, and improve diagnostic stability while retaining a traceable reasoning process. To achieve this objective, CD-IBRB integrates XGBoost-based feature selection, Kendall-rank-correlation-guided attribute decoupling, attribute-wise interval rule construction, reliability-adjusted evidential reasoning, and constrained P-CMA-ES parameter optimization.

Within the investigated experimental scope, CD-IBRB achieved an accuracy of 0.9702 and a macro-averaged F1 score of 0.9703, exceeding the strongest BRB variant and data-driven baseline by 7.70 and 6.10 percentage points, respectively. The ablation experiments verified the separate contributions of attribute decoupling and parameter optimization, while the cross-load, disturbance, and benchmark experiments demonstrated its adaptability under the evaluated operating-condition variations, input disturbances, and heterogeneous classification tasks. The principal scientific novelty lies in the coordinated integration of correlation-aware attribute transformation and additive attribute-wise interval rules within an uncertainty-aware BRB framework. This design reduces rule-base growth while preserving explicit rule activation, belief representation, and evidential aggregation. These findings indicate that the primary research objective was achieved within the tested conditions and that CD-IBRB provides a promising decision-support approach for interpretable bearing fault diagnosis. Its broader industrial applicability, particularly under severe domain shifts, transient operating processes, compound faults, and continuously evolving degradation, requires further validation using multisensor and field-scale experiments.

## Figures and Tables

**Table 1:** Experimental data for bearing fault diagnosis.

Number	Speed	Load	Normal	ORF	IRF	BF
Training set samples	2000 r/min	0 N	344	344	344	344
Test set samples		20 N	344	344	344	344

**Table 2:** Initial parameter table.

ID	Referential Interval	Rule Reliability	Rule Weight	Output
1	[−0.7197, −0.4707]	1	1	{0.0284, 0.0071, 0.0071, 0.9574}
2	[−0.4707, −0.3846]	1	1	{0.4326, 0.0071, 0.0355, 0.5248}
3	[−0.3846, −0.3153]	1	1	{0.4930, 0.0070, 0.0986, 0.4014}
4	[−0.3153, −0.2328]	1	1	{0.4648, 0.0141, 0.1268, 0.3944}
5	[−0.2328, −0.0957]	1	1	{0.4326, 0.0426, 0.3404, 0.1844}
6	[−0.0957, 0.0382]	1	1	{0.3901, 0.2128, 0.3901, 0.0071}
7	[0.0382, 0.2036]	1	1	{0.2254, 0.3662, 0.4014, 0.0070}
8	[0.2036, 0.3886]	1	1	{0.0213, 0.6525, 0.3191, 0.0071}
…	…	…	…	…
70	[0.5122, 3.8255]	1	1	{0.0070, 0.5704, 0.4155, 0.0070}

**Table 3:** Main parameter settings of P-CMA-ES.

Initial Step Size	Offspring Population Size	Number of Selected Offspring	Maximum Generations	Evolution-Path Coefficient $c_{c}$	Rank-One Coefficient $c_{1}$	Rank-Multiple Coefficient $c_{\mu }$
0.03	22	11	200	0.00947	$1.13\times 10^{-5}$	$5.21\times 10^{-5}$

**Table 4:** Optimized parameters of the CD-IBRB model.

ID	Referential Interval	Rule Reliability	Rule Weight	Output
1	[−0.7197, −0.4707]	1	0.8162	{0.0743, 0.0911, 0.0292, 0.8054}
2	[−0.4707, −0.3846]	0.9198	0.9588	{0.3132, 0.0556, 0.2173, 0.4139}
3	[−0.3846, −0.3153]	0.9328	0.9753	{0.3549, 0.0342, 0.2149, 0.3960}
4	[−0.3153, −0.2328]	0.6944	0.9376	{0.3165, 0.0000, 0.1676, 0.5158}
5	[−0.2328, −0.0957]	0.8595	0.8175	{0.2628, 0.1139, 0.5141, 0.1092}
6	[−0.0957, 0.0382]	0.8545	0.7365	{0.2401, 0.2123, 0.5421, 0.0055}
7	[0.0382, 0.2036]	0.9852	0.9833	{0.3568, 0.3286, 0.3121, 0.0026}
8	[0.2036, 0.3886]	0.7317	0.9732	{0.0226, 0.5217, 0.4260, 0.0296}
…	…	…	…	…
70	[0.5122, 3.8255]	0.8862	0.8502	{0.0207, 0.5162, 0.2998, 0.1633}

**Table 5:** Class-wise diagnostic performance of the CD-IBRB model.

Model	Accuracy	Macro F1 Score	Normal		ORF		IRF		BF	
			Precision	Recall	Precision	Recall	Precision	Recall	Precision	Recall
CD-IBRB	0.9702	0.9702	0.941	0.9738	0.9912	0.9912	0.985	0.9593	0.9648	0.9563

**Table 6:** Ablation results of CD-IBRB and comparison with the conventional BRB.

Model	Accuracy	Macro F1 Score	Normal		ORF		IRF		BF	
			Precision	Recall	Precision	Recall	Precision	Recall	Precision	Recall
CD-IBRB	0.9702	0.9702	0.941	0.9738	0.9912	0.9912	0.985	0.9593	0.9648	0.9563
CD-IBRB0	0.9053	0.9046	0.9226	0.8662	0.8936	0.904	0.8901	0.9418	0.9149	0.9069
CD-IBRB1	0.9303	0.9302	0.921	0.9156	0.9376	0.9186	0.9235	0.9476	0.9389	0.9389
IBRB	0.8506	0.85	0.843	0.8297	0.8529	0.843	0.8558	0.8459	0.8768	0.8691
BRB	0.9149	0.9147	0.9063	0.9563	0.9057	0.9215	0.9325	0.8837	0.9169	0.8982

**Table 7:** Detailed bearing fault diagnosis performance of different models.

Model	Accuracy	Macro F1 Score	Normal		ORF		IRF		BF	
			Precision	Recall	Precision	Recall	Precision	Recall	Precision	Recall
CD-IBRB	0.9702	0.9703	0.941	0.9738	0.9912	0.9912	0.985	0.9593	0.9648	0.9563
ABRB	0.835	0.8351	0.8511	0.8411	0.8022	0.8255	0.85	0.8401	0.8381	0.843
EBRB	0.8932	0.8932	0.9117	0.9011	0.9069	0.9069	0.8724	0.875	0.8818	0.8895
HBRB	0.8503	0.8503	0.8604	0.8604	0.8532	0.8284	0.8419	0.8517	0.8457	0.8604
DT	0.9092	0.9067	0.8171	1	0.9364	0.9419	0.9044	0.7151	1	0.9797
BPNN	0.8714	0.8715	0.7929	0.8459	0.9715	0.9912	0.9082	0.8343	0.8187	0.8139
LR	0.8081	0.8073	0.7456	0.75	0.9241	0.9563	0.8566	0.6947	0.724	0.8313
NB	0.7573	0.7572	0.6195	0.7005	0.9005	0.9215	0.8781	0.6075	0.6926	0.7994

**Table 8:** Quantitative comparison of different models in cross-load experiments.

Model	Accuracy ± SD	Macro F1 ± SD	Macro Precision ± SD	Macro Recall ± SD
CD-IBRB	0.8612 ± 0.1531	0.8607 ± 0.1539	0.8619 ± 0.1536	0.8612 ± 0.1531
ABRB	0.7107 ± 0.1651	0.6939 ± 0.1704	0.7032 ± 0.1737	0.7107 ± 0.1651
EBRB	0.7873 ± 0.1809	0.7732 ± 0.1856	0.781 ± 0.1881	0.7879 ± 0.1809
HBRB	0.7427 ± 0.1736	0.7275 ± 0.1794	0.7358 ± 0.1817	0.7427 ± 0.1736
DT	0.7449 ± 0.239	0.6992 ± 0.316	0.6978 ± 0.3649	0.7449 ± 0.239
BPNN	0.7747 ± 0.3086	0.7365 ± 0.3748	0.7194 ± 0.4089	0.7747 ± 0.3086
LR	0.7674 ± 0.2476	0.7209 ± 0.3268	0.7042 ± 0.3642	0.7674 ± 0.2476
NB	0.6734 ± 0.1719	0.6135 ± 0.2415	0.6689 ± 0.3317	0.6734 ± 0.1719

**Table 9:** Quantitative performance comparison of different models under varying disturbance intensities.

Model	Accuracy					
	Disturbance Intensity: 0.001	Disturbance Intensity: 0.005	Disturbance Intensity: 0.01	Disturbance Intensity: 0.02	Disturbance Intensity: 0.05	Disturbance Intensity: 0.1
CD-IBRB	0.9767	0.9767	0.9767	0.9767	0.9688	0.9571
ABRB	0.9419	0.9433	0.9426	0.939	0.9288	0.9077
EBRB	0.9528	0.9513	0.9535	0.9499	0.939	0.9186
HBRB	0.9491	0.9506	0.9499	0.9455	0.9346	0.9135
DT	0.96	0.9578	0.9608	0.952	0.9302	0.8953
BPNN	0.9666	0.9651	0.9673	0.9593	0.9397	0.9077
LR	0.9688	0.9673	0.9688	0.9608	0.9433	0.9121
NB	0.9651	0.9666	0.9637	0.9557	0.9331	0.8993

**Table 10:** Representative activated rule for an inner-ring fault sample.

Sample Information	Activated Rule Number	Rule Antecedent	Rule Reliability	Normalized Rule Weight	Consequent Belief Distribution
True class: IRF; predicted class: IRF	Rule 56	$X_{7}^{*}\in \left[0.6389,1.4780\right]$	1	0.9058	{NC: 0.1293, IRF: 0.7950, BF: 0.0039, ORF: 0.0718}

**Table 11:** Computational complexity of CD-IBRB and the comparison models.

Stage	Inference Complexity per Sample
Attribute decoupling	$O!\left(E_{Q}\left(PM^{2}+nM^{2}\right)\right)$
P-CMA-ES optimization	$O!\left(G[\lambda nMC+\lambda D^{2}+D^{3}]\right)$
Single-sample reasoning	$O!\left(M^{2}+M\left(K+C\right)\right)$

## Data Availability

The bearing fault data analyzed in this study were obtained from the publicly available Shandong University of Science and Technology bearing dataset (SDUST Dataset, https://github.com/JRWang-SDUST/SDUST-Dataset.git; accessed on 18 August 2026). The benchmark datasets used in the generalization experiments are available through the UCI Machine Learning Repository, http://archive.ics.uci.edu/ml/index.php (accessed on 18 August 2026), and the KEEL Dataset Repository, https://sci2s.ugr.es/keel/datasets.php (accessed on 18 August 2026). All datasets used in this study are publicly accessible through these repositories.

## Appendix A. Characteristics and Sample Distributions of the Benchmark Datasets

**Table A1:** Characteristics and Sample Distributions of the Benchmark Datasets.

Datasets	Number of Classes	Size	Distribution	Number of Attributes
Ecoli	8	336	143/77/52/35/20/5/2/2	7
Iris	3	150	50/50/50	4
New-Thyroid	3	215	150/35/30	5
Shuttle	7	14,500	11,478/13/39/2155/809/4/2	9
Abalone	4	4177	1407/2077/557/136	8

## Appendix B. Detailed Performance Metrics for the Generalization Experiments

**Table A2:** Detailed Performance Metrics for the Generalization Experiments.

Datasets	Models	Accuracy	Macro F1-Score	Macro Precision	Macro Recall
Ecoli	CD-IBRB	0.9083	0.8699	0.8687	0.9083
	ABRB	0.794	0.7579	0.7548	0.794
	EBRB	0.8379	0.8069	0.8051	0.8379
	HBRB	0.8212	0.7897	0.7871	0.8212
	DT	0.7921	0.7848	0.7885	0.7921
	BPNN	0.7921	0.5217	0.5138	0.5805
	LR	0.8119	0.5401	0.543	0.589
	NB	0.7426	0.4942	0.5356	0.5746
New-Thyroid	CD-IBRB	0.9895	0.9701	0.9713	0.9688
	ABRB	0.9082	0.8919	0.8878	0.8961
	EBRB	0.9259	0.908	0.9112	0.9049
	HBRB	0.9373	0.922	0.9222	0.9218
	DT	0.9538	0.9509	0.9567	0.9538
	BPNN	0.9385	0.9108	0.9728	0.8653
	LR	0.9231	0.8825	0.9667	0.8283
	NB	0.9077	0.8634	0.9608	0.8047
Iris	CD-IBRB	0.9618	0.9561	0.9585	0.9538
	ABRB	0.8512	0.8481	0.8458	0.8504
	EBRB	0.9079	0.9024	0.9025	0.9024
	HBRB	0.8839	0.8764	0.8754	0.8774
	DT	0.9333	0.9327	0.9444	0.9333
	BPNN	0.9111	0.9095	0.9298	0.9111
	LR	0.8444	0.8443	0.8452	0.8444
	NB	0.9111	0.9107	0.9155	0.9111
Shuttle	CD-IBRB	0.9696	0.9413	0.9414	0.9411
	ABRB	0.9257	0.8993	0.9029	0.8957
	EBRB	0.941	0.9118	0.9131	0.9106
	HBRB	0.9485	0.9241	0.9229	0.9254
	DT	0.997	0.9968	0.9968	0.997
	BPNN	0.9942	0.7853	0.8211	0.7603
	LR	0.9168	0.3833	0.3956	0.3754
	NB	0.8628	0.5921	0.6004	0.7779
Abalone	CD-IBRB	0.673	0.4246	0.4809	0.4225
	ABRB	0.6098	0.5857	0.5904	0.5812
	EBRB	0.636	0.6113	0.6156	0.6071
	HBRB	0.6189	0.5949	0.594	0.5957
	DT	0.6507	0.4317	0.4989	0.4217
	BPNN	0.6834	0.4827	0.5884	0.4655
	LR	0.6691	0.4092	0.4504	0.4143
	NB	0.6037	0.416	0.4742	0.4132
